# Carbazole–Phosphazene Based Polymer for Efficient Extraction of Gold and Precious Elements from Electronic Waste

**DOI:** 10.1021/acsomega.4c09068

**Published:** 2024-11-18

**Authors:** Evren Cucu, Betul Ari Engin, Murat Tunc, Ramazan Altundas, Ali Enis Sadak

**Affiliations:** 1 TUBITAK UME, Chemistry Group Laboratories, 41470, Gebze, Kocaeli, Türkiye; 2 Department of Chemistry, Gebze Technical University,, 41400, Gebze, Kocaeli, Türkiye

## Abstract

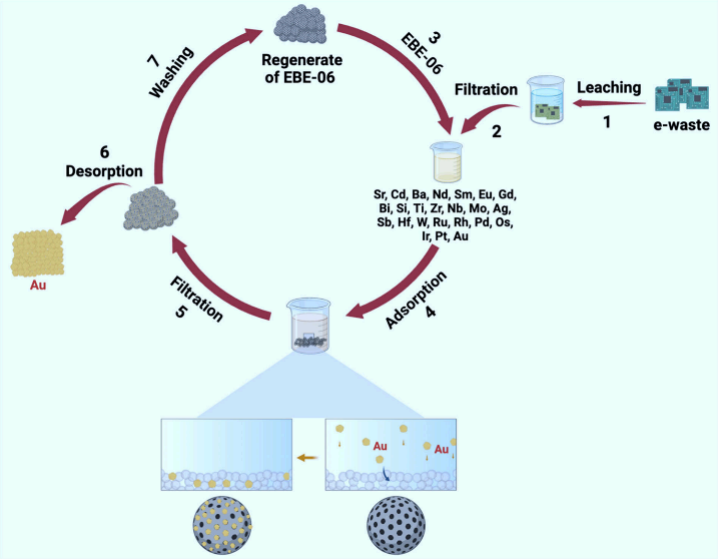

The continuous advancement of industry and technology has significantly increased electronic waste, which contributes to the depletion of valuable metal reserves. Therefore, it is crucial to recycle precious metals in electronic waste effectively and sustainably. This study introduces a novel approach by applying a carbazole–phosphazene-based polymer, EBE-06, in a two-stage leaching method for efficient metal extraction. In the first leaching stage, tin is selectively separated using an acid solution at a controlled pH. In the second stage, valuable metals such as gold are recovered through adsorption onto EBE-06. The polymer exhibited a 99% gold adsorption rate within 1 h, independent of pH, and a maximum adsorption capacity of 1.787 g of gold per gram of polymer. The desorption process yielded 95% efficiency, with the polymer maintaining 94% efficiency over three cycles of use.

## 1 Introduction

The rapid advancement of technology has significantly increased the use of electrical and electronic equipment (EEE), making it indispensable in modern life. However, the frequent replacement of devices, driven by technological obsolescence, has resulted in a dramatic rise in electronic waste (e-waste), reaching approximately 53.6 million tons globally in 2019.^1−4^ Printed circuit boards (PCBs), a major component of e-waste, contain up to 40% metal by weight, with precious metals like gold often present in higher concentrations than in natural ores.^5−7^ Therefore, the efficient recovery of these valuable metals presents both an economic opportunity and an environmental necessity.

Among the methods developed for metal recovery, pyrometallurgy, hydrometallurgy, and biometallurgy are the most prominent. Pyrometallurgy, which relies on high-temperature processes to extract metals, is effective but environmentally harmful due to its carbon emissions.^8^ Hydrometallurgy offers selective metal recovery through chemical leaching, but many adsorbents used in this process degrade under acidic conditions or lose functionality after a single use.^9−29^ Biometallurgy, which employs microorganisms for metal extraction, remains in its infancy and is not yet viable for industrial-scale applications.^30−32^ Despite their potential, current technologies are limited by inefficiencies in selectivity, capacity, and stability, particularly under the harsh conditions required for metal extraction from PCBs.

**Figure 1:**
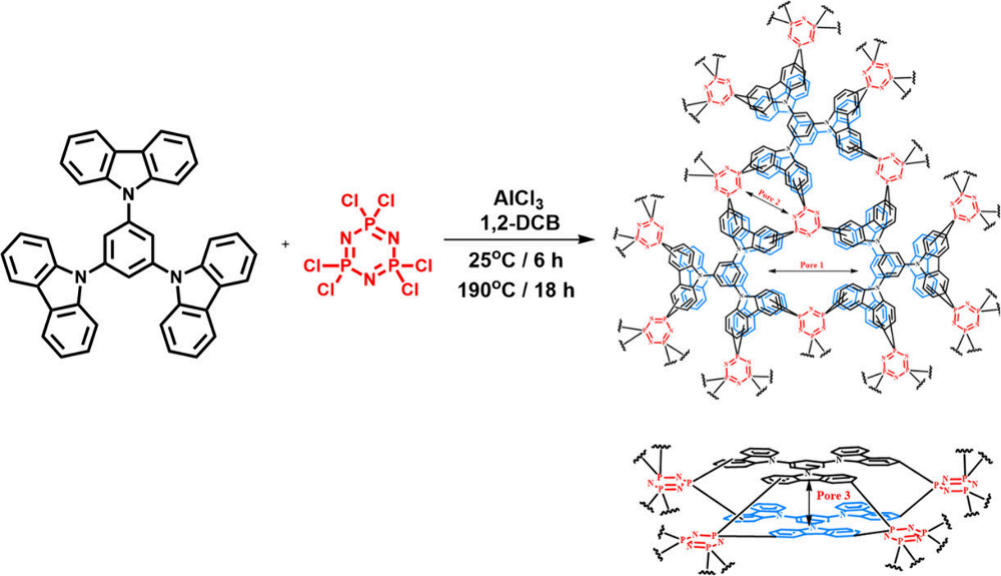
Synthesis and proposed structure of EBE-06.

This study addresses these challenges by introducing a novel carbazole–phosphazene-based polymer (EBE-06) synthesized by Sadak et al.^33^ with a unique structure designed for selective metal adsorption. EBE-06 exhibits remarkable stability in acidic environments and high adsorption capacities, particularly for gold, with a demonstrated ability to maintain a performance over multiple cycles (Figure 1). Unlike traditional adsorbents, this polymer’s high heteroatom content and porous structure enable it to selectively capture precious metals, providing a sustainable and scalable solution for e-waste recycling.^21^ Through the development of a two-stage leaching process, we provide a more efficient method for the recovery of gold and other valuable metals from PCBs, advancing the current state of hydrometallurgical recycling techniques.^34−43^

## 2 Materials and Methods

All solvents and chemicals were used as purchased without extra distillation or purification. 1,2-Dichlorobenzene ≥98% and tetrahydrofuran ACS reagent (99.8%) purities were purchased from Merck. AlCl_3_ anhydrous ≥99% purity was purchased from Fluka. 1,3,5-Tri(9*H*-carbazol-9-yl) benzene ≥98% and hexachlorocyclotriphosphazene >95% purities were purchased from Chemenu China. Methanol ACS reagent (99.8%) purities were purchased from Sigma-Aldrich. Acetone analytical grade (99.5%) was purchased from J. T. Baker. Nitric acid (HNO_3_) > 65% purities were purchased from isolab, and hydrochloric acid (HCl) > 35% purities were purchased from Sigma-Aldrich. Sulfuric acid (H_2_SO_4_) 95–97% purities were purchased from Merck, and thiourea (CS(NH_2_)_2_) ACS reagent (≥99%) purities were purchased from Sigma-Aldrich. The gold capture and separation ability of EBE-06 were verified by scanning and transmission electron microscopy (SEM and STEM), energy-dispersive X-ray spectroscopy (EDX), powder X-ray diffraction (PXRD), X-ray photoelectron spectroscopy (XPS), inductively coupled plasma mass spectrometry (ICP-MS), and Brunauer–Emmett–Teller (BET).

### 2.1 Synthesis of EBE-06

Cyclophosphozene (hexachlorocyclotriphosphazene) and tricarbazole (1,3,5-tri(9*H*-carbazol-9-yl)benzene) based porous polymer (Figure 1) called EBE-06 was synthesized according to the previous study.^33^ Anhydrous AlCl_3_ (2.79 g, 20.92 mmol, 12 equiv) was added in 40 mL of 1,2-dichlorobenzene at room temperature, and this mixture was stirred for 15 min. Then hexachlorocyclotriphosphazene (606 mg, 1.74 mmol, 1 equiv) in 15 mL of 1,2-dichlorobenzene and 1,3,5-tri(9*H*-carbazol-9-yl) benzene (1.00 g, 1.74 mmol, 1 equiv) in 20 mL of 1,2-dichlorobenzene were added to this mixture and stirred for 6 h at room temperature. After 6 h, it was heated to 190 °C and stirred for 18 h. The reaction mixture was cooled to room temperature. The black solids were filtered through a No. 3 glass filter and washed subsequently with 200 mL of 2 M HCl, 200 mL of distilled water, and 100 mL of methanol. The crude material in 100 mL of methanol was sonicated for 30 min and it was filtered; solid was sequentially extracted by Soxhlet with methanol, tetrahydrofuran, and acetone (100 mL for each cycle) for 24 h. After the washing processes were completed, the material was dried in a vacuum oven at 50 °C for 6 h, at 70 °C for 6 h, and at 120 °C for 24 h. The EBE-06 was obtained as a brown solid in 1.198 g, 98% yield. IR (ATR, powder): 1590, 1454, 1311, 1214, 1056, 867, 803, 589, 541 cm^–1^. The surface area was examined by BET analysis before the metal adsorption study (Table 1).

**Table 1:** Textural Properties of EBE-06 and Au Loaded EBE-06

Polymer	*S*_BET_^a^ [m^2^ g^–1^]	*S*_Lang_^b^ [m^2^ g^–1^]	*S*_micro_^c^ [m^2^ g^–1^]	*V*_t_^d^ [cm^3^ g^–1^]	*V*_0.1_^e^ (cm^3^ g^–1^)	%*V*_0.1_/*V*_t_^f^	%*S*_micro_/*S*_BET_	4*V*/*A*_BET_^g^ [nm]
**EBE-06**	1987	2307	1437	0.9512	0.7859	82.62	72.32	1.9149
EBE-06 **Au Loaded**	654	723	277	0.3296	0.2710	82.22	42.35	2.0162

a BET surface area calculated from the N_2_ adsorption isotherm in the relative pressure (*P*/*P*_0_) range from 0.01 to 0.10.

b Langmuir surface area calculated from the N_2_ adsorption isotherm in the pressure range from 30 to 220 mbar.

c Micropore surface area calculated from the N_2_ adsorption isotherm using the t-plot method from the Harkins–Jura equation.

d Total pore volume at *P*/*P*_0_ = 0.99.

e Micropore volume at *P*/*P*_0_ = 0.1.

f Microporosity ratio.

g Average pore diameter.

### 2.2 Metal Selectivity Experiments

For the metal selectivity analysis of EBE-06, three different standard solutions containing 48, 13, and 7 elements with a concentration of 100 ppm were diluted to a concentration of 100 ppb along with adjusting the pH to 2. To minimize experimental error, tubes were arranged in two groups, one with the polymer and the other without the polymer, and each group was taken in a shaker incubator under the same conditions. To each experimental sample, 10 mg of EBE-06 was added, and the mixture was magnetically stirred at 120 rpm at room temperature for 24 h. After 24 h, each sample was filtered using a 0.45 μm hydrophobic membrane polytetrafluoroethylene (PTFE) filter using a 3 mL plastic syringe and the metal concentrations in each group were measured using ICP-MS (Table S1). The metal selectivity of EBE-06 was obtained by averaging the concentrations of control samples and parallel measurement results through the following equation, where *Ck* is the average concentration of control samples and *Cd* is the average concentration of experimental samples:
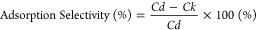
1

### 2.3 Maximum Adsorption Capacity Experiments of Gold Ions

A 10.000 ppm solution of gold(III) chloride trihydrate at pH 2 was prepared by using deionized water and HCl to determine the maximum gold adsorption capacity of EBE-06. The stock solution used in gold adsorption experiments was prepared previously by dissolving 1.307 g of gold(III) chloride trihydrate (HAuCl_4_·3H_2_O) in 65 mL of pH 2 with deionized water and HCl. The solution was left to stabilize for 7 days at +4 °C. After 7 days, it was filtered using a 0.45 μm PTFE filter. Necessary dilutions were made using a 2% HNO_3_ solution, and the concentration value was determined by ICP-MS. The gold solutions of 20, 100, 250, 500, 750, 1000, 3000, 5000, and 7500 ppm were obtained by using the resulting stock solution with pH 2 water and HCl solution. 10 mL of each solution was taken for three parallel measurements. 10 mg of EBE-06 was added to each of these solutions. The mixtures were stirred for 48 h, and then the solutions were filtered. Subsequently, the solutions of the filtrates were measured using ICP-MS, and the remaining amount of gold ions in each solution was calculated.

## 3 Results and Discussion

### 3.1 Metal Selectivity under pH Range

**Figure 2:**
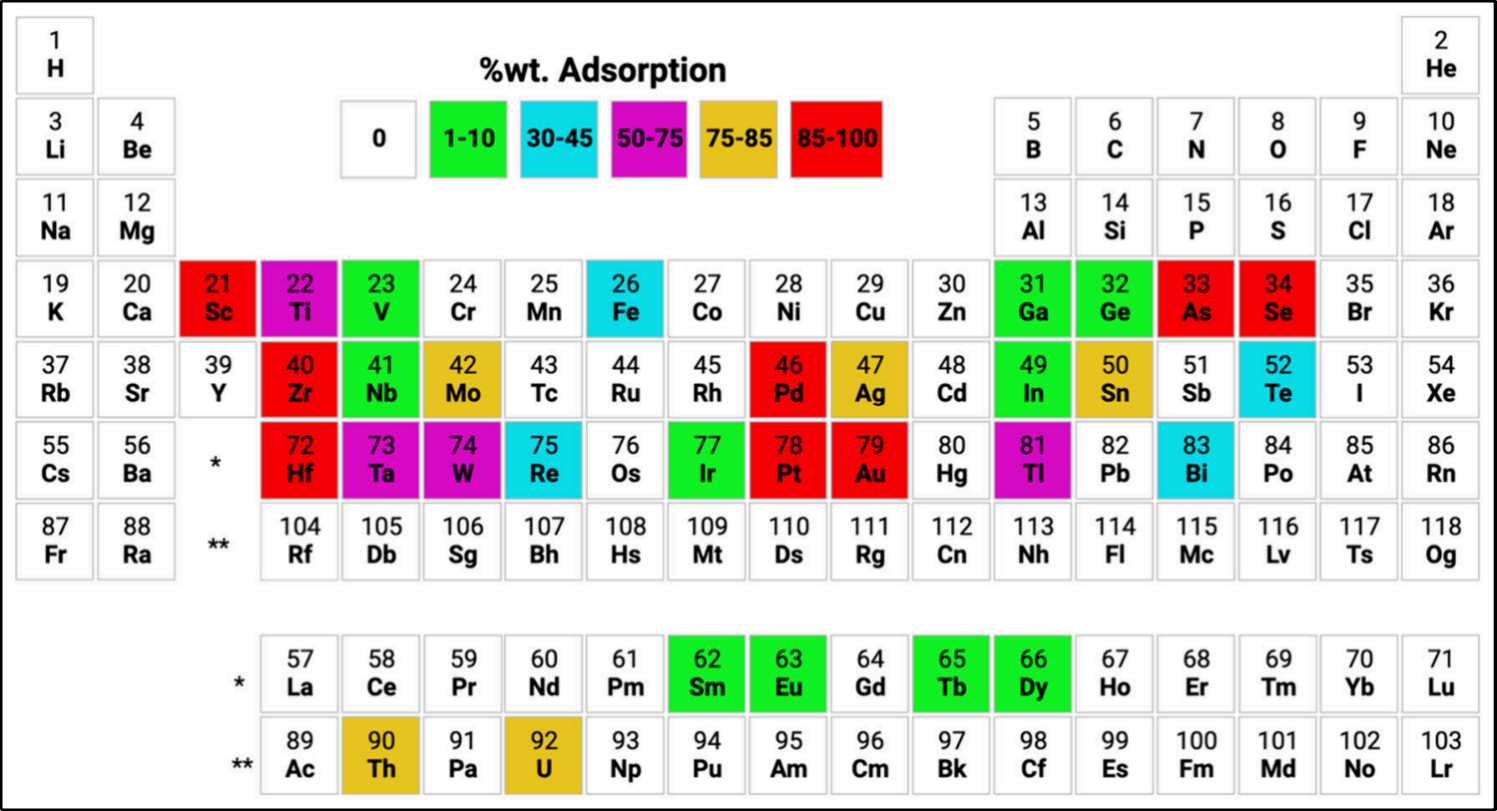
Periodic table of %wt adsorption values from selectivity experiments. (For the actual adsorption values, please see Table S1.)

**Figure 3:**
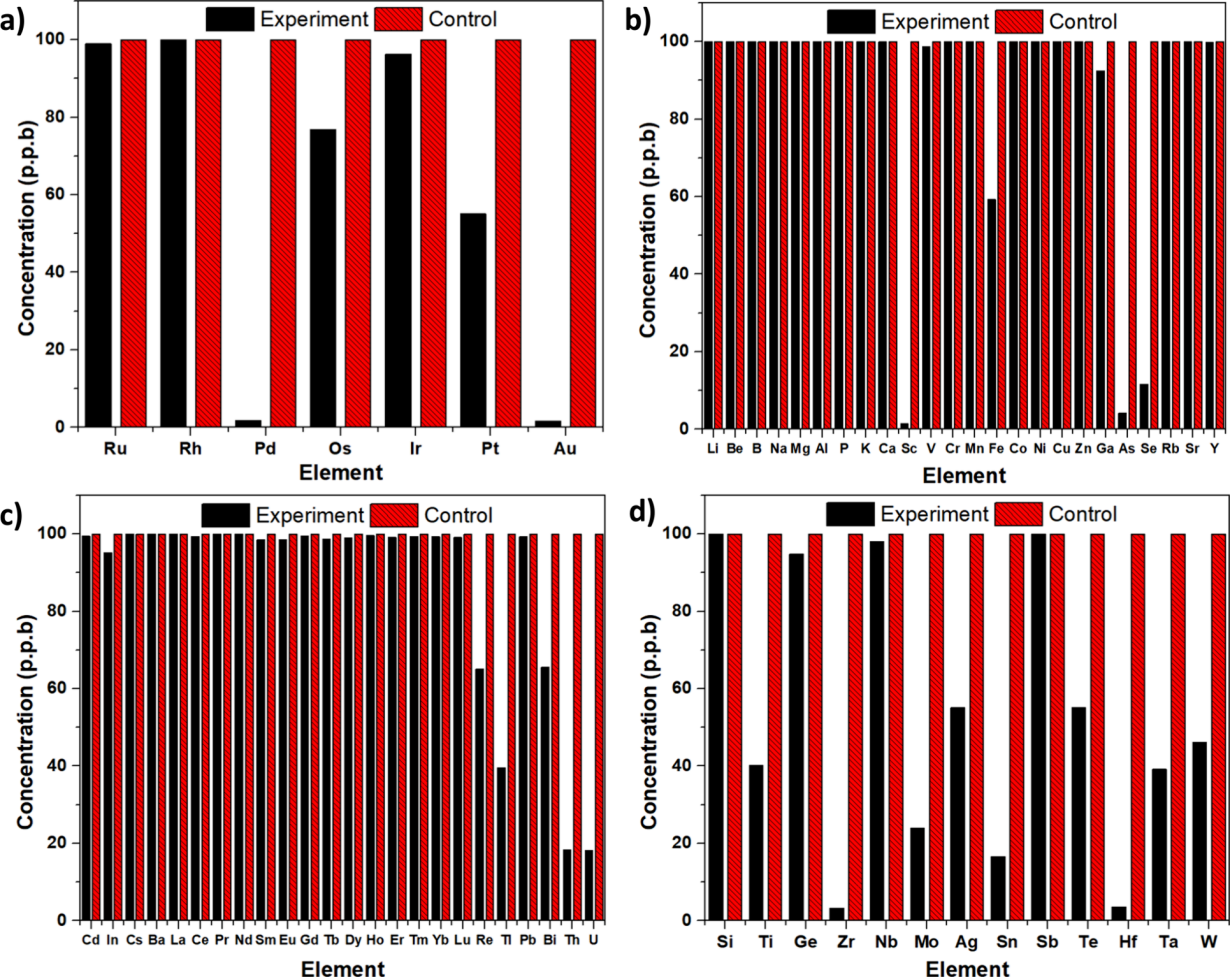
(a) Adsorption selectivity of EBE-06 in the first standard solution, (b) adsorption selectivity of EBE-06 in the first part of the second standard solution, (c) adsorption selectivity of EBE-06 in the second part of the second standard solution, and (d) adsorption selectivity of EBE-06 in the third standard solution.

The periodic table provided in Figure 2 and Figure 3a–d shows the % adsorption values for adsorption experiments conducted in three different standard solutions. The periodic table provided in Figure 2 notably indicates high adsorption values for Au (98.47%), Pd (98.18%), Pt (45.00%), Sc (98.63%), Fe (40.74%), As (95.88%), Se (88.43%), Tl (60.48%), Th (81.79%), and U (81.80%). It is essential to know that, despite the EBE-06 material exhibiting high selectivity against ions such as Sc, Se, Tl, Th, and U, these elements are not found in PCBs.

Additionally, data for the specific adsorption values are provided in Table S1. Based on the data obtained from the selectivity experiment, it was found that EBE-06 exhibits high adsorption and selectivity capacities for valuable metals such as Au, Ag, Pt, and Pd. To assess the influence of pH changes on the adsorption rate and capacity of precious metals in EBE-06 solutions, each containing approximately 50 ppb gold, platinum, silver, and palladium ions in aqueous solutions were prepared with pH values of 2, 4, 7, and 9. The concentration of metal ions in each solution was measured by ICP-MS at 1, 2, 3, 6, 12, and 24 h, and the adsorption efficiency was calculated by comparing it with the initial concentrations.

**Figure 4:**
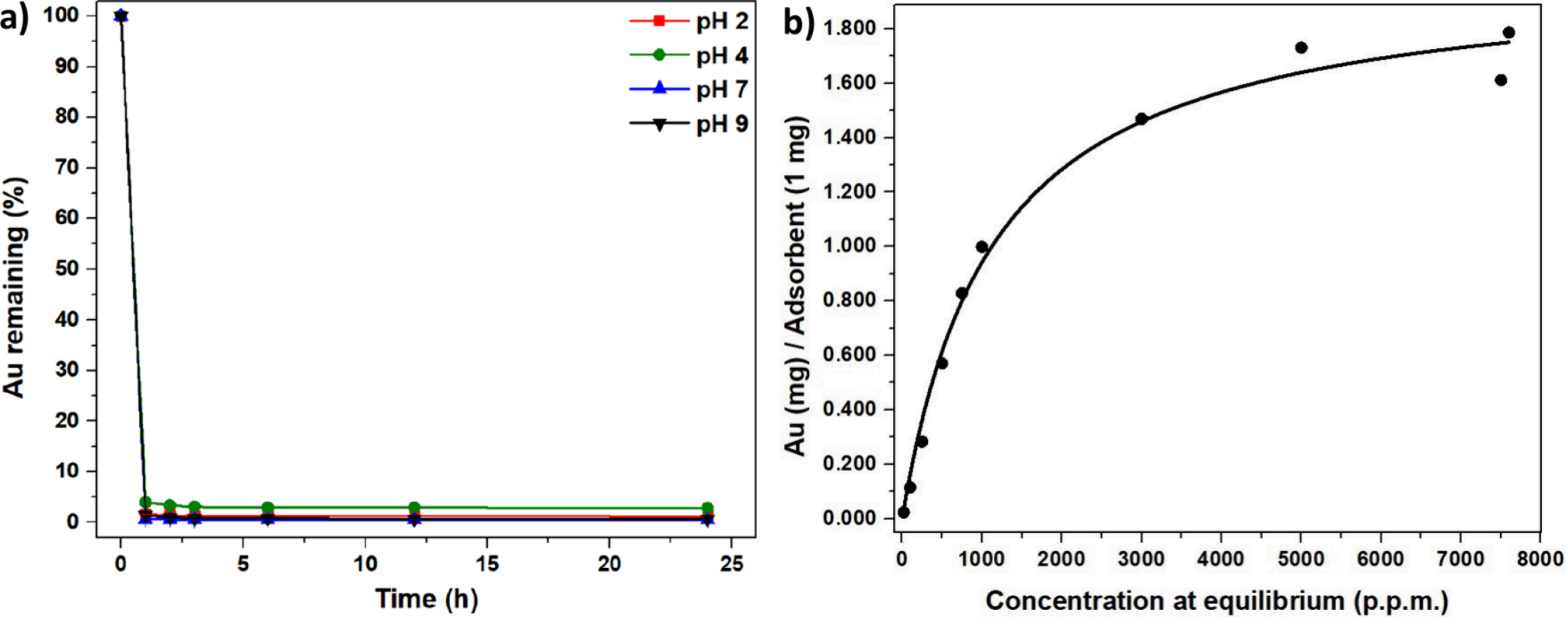
(a) Time-dependent gold adsorption efficiencies at varying pH values. (b) Langmuir plot isotherm of EBE-06 from the gold adsorption experiment.

The average of the obtained data was analyzed for gold ion adsorption at varying pH levels. The measurements at four different pH values revealed that EBE-06 achieved adsorption close to 100% within the first hour at all pH levels, indicating the rapid adsorption of gold ions (Figure 4a). Examining platinum ion adsorption with varying pH, the highest adsorption was observed at pH 4, reaching approximately 93% after 24 h (Figure S1). For silver ions, the highest adsorption, around 80%, occurred at pH 2 after 24 h (Figure S2). Regarding palladium ion adsorption, the highest adsorption rate was observed, roughly 100%, at pH 7 after 24 h (Figure S3).

### 3.2 Measuring the Maximum Adsorption Capacity of Gold Ions

The Langmuir plot from the measurement obtained shows that EBE-06 polymer can adsorb 1.787 mg of gold per milligram of polymer (Figure 4b).

**Figure 5:**
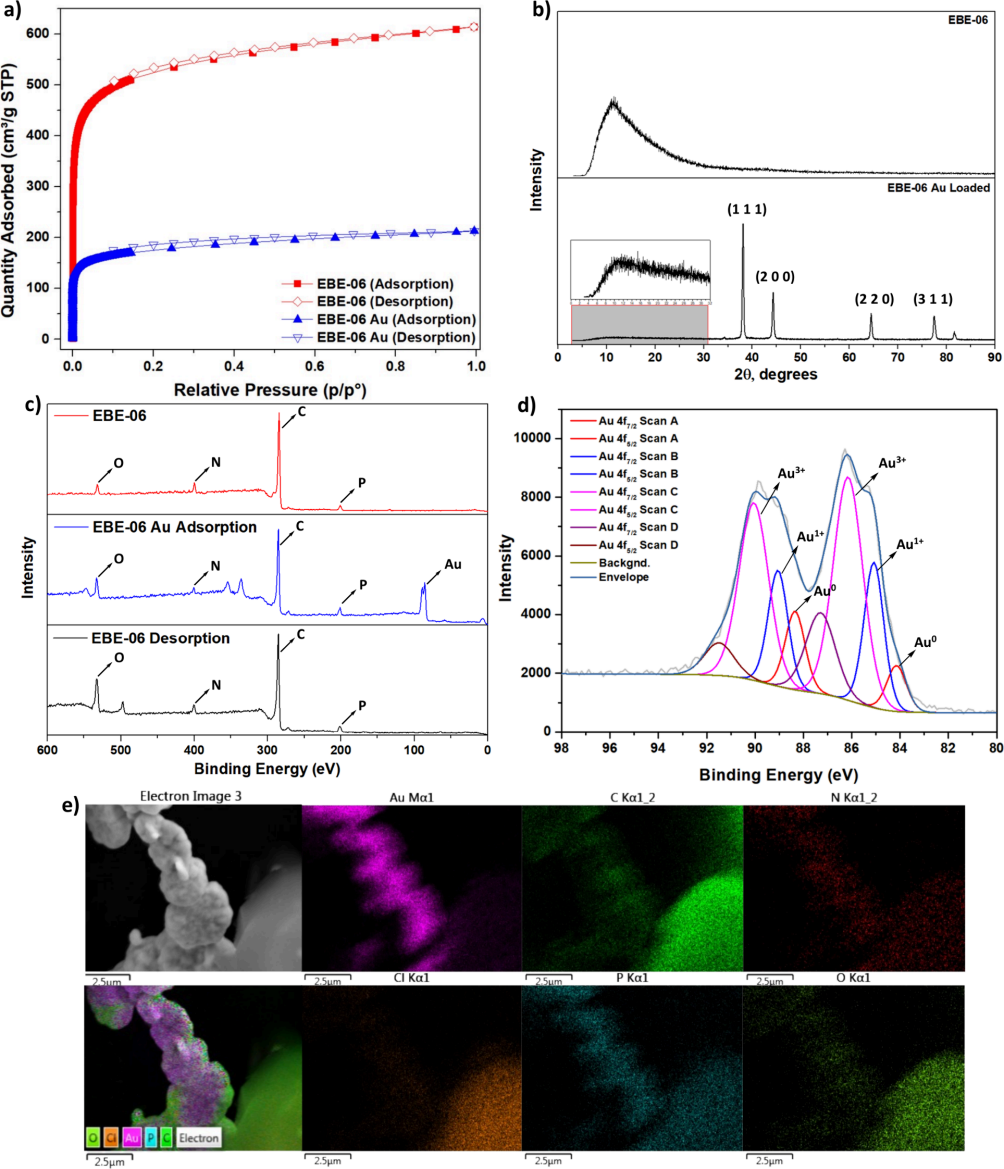
(a) Nitrogen adsorption–desorption isotherms of EBE-06 and EBE-06 Au measured at 77 K. (b) XRD patterns for EBE-06 and EBE-06 Au loaded. (c) XPS spectra of EBE-06, EBE-06 Au adsorption, and EBE-06 desorption. (d) XPS spectra Au (4 f) of Au loaded EBE-06. (e) EDX images of Au loaded EBE-06 (for more images, see Figure S8).

The BET surface area analysis was performed to determine changes in the surface area of gold-loaded EBE-06. The results showed a significant reduction in the surface area of EBE-06 (Figure 5a, Table 1). The XRD spectrum, confirming the capability of EBE-06 to capture gold ions, revealed 2θ angles (1 1 1), (2 0 0), (2 2 0), and (3 1 1) indicating the presence of gold ions (Figure 5b).^37,38^ The XPS spectrum of gold-loaded EBE-06 shows signals between 82 and 94 eV (Figure 5c). Through detailed scanning, the oxidation states of gold ions were determined as gold 0 at 84.14 (4f7/2) and 88.33 (4f5/2) eV, +1 at 85.08 (4f7/2) and 89.03 (4f5/2) eV, and +3 at 86.15 (4f7/2) and 90.05 (4f5/2) eV (Figure 5d).^39−41^ The XPS spectrum obtained after desorption processes verifies the disappearance of gold signals; it confirms that the desorption was completed (Figure 5c). Additionally, elemental mapping conducted with EDX analysis indicates the abundant presence of gold in EBE-06 (Figure 5e, Figure S8).

### 3.3 Determining the Desorption Conditions

**Figure 6:**
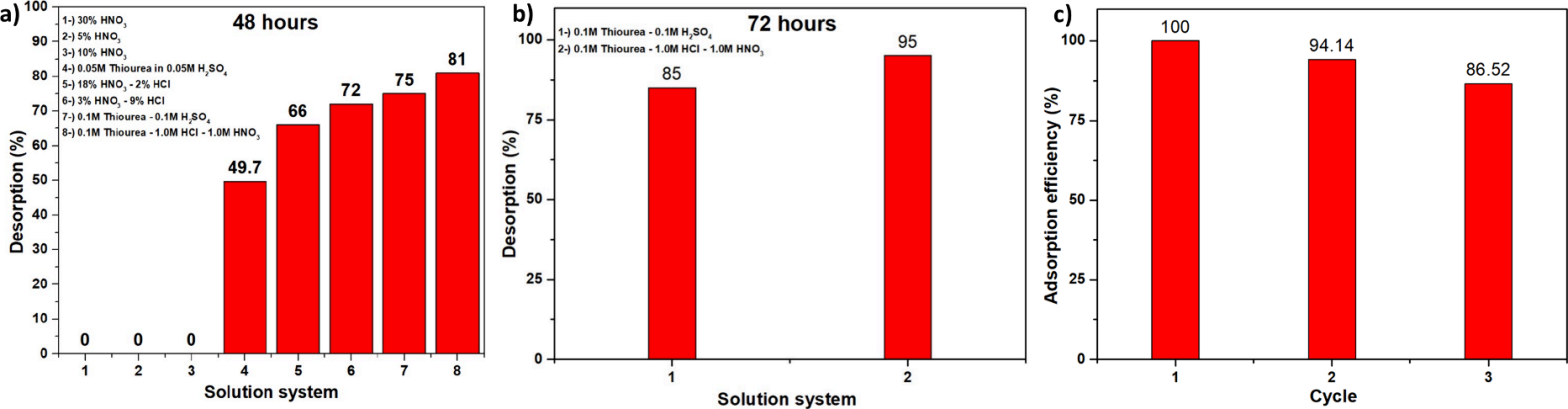
(a) Desorption efficiencies in different solutions and at 48 h. (b) Desorption efficiencies at 72 h. (c) Gold capture efficiencies for three consecutive adsorption–desorption cycles.

Solutions for determining desorption conditions were prepared by mixing HNO_3_, thiourea, H_2_SO_4_, HCl, and deionized water in specified proportions, as indicated in previous studies.^19−21^ These solutions were made using a ratio of 1 mL per 1 mg of the gold-loaded polymer. The mixtures were stirred at 60 °C for 48 h. After 48 h, the concentration of desorbed gold ions in each sample was determined using ICP-MS. The results indicated that the solution mixture of 0.1 M thiourea/1 M HCl/1 M HNO_3_ had the highest desorption efficiency at 81% (Figure 6a). In the second stage, a second 72 h experiment was conducted for these two experiments. In the second stage, 0.1 M thiourea/0.1 M H_2_SO_4_ and 0.1 M thiourea/1.0 M HCl/1.0 M HNO_3_ solution systems were used again. The polymer adsorbed with gold was added to these solutions (1 mL of solution for each milligram of polymer), and the prepared solutions were mixed at 60 °C for 72 h at 120 rpm. After 72 h, the concentration of gold ions desorbed in each experimental sample was determined by ICP-MS. The results obtained after 72 h (Figure 6b, Figure S9 for EDX images) showed that the mixture of 0.1 M thiourea/1.0 M HCl/1.0 M HNO_3_ had the highest desorption efficiency of approximately 95 ± 6%.

### 3.4 Regeneration of EBE-06

To determine the reusability of EBE-06, after the desorption process, the gold desorbed material was refluxed with a 2% NaOH solution for 18 h, washed with deionized water, and then dried in a vacuum oven at 90 °C before being reused for adsorption. The adsorption process was then applied and the remaining gold ion concentration in the solution was measured using the ICP-MS. Three times repeated adsorption–desorption experimental results revealed that EBE-06 material has an adsorption efficiency of 100% in the first cycle, 94% in the second cycle, and 87% in the third cycle (Figure 6c).

### 3.5 Precious Metal Capture from Actual PCB e-Waste

The applicability of the EBE-06 polymer for the adsorption and recovery of precious metals, particularly gold, from electronic waste was demonstrated through a series of experiments conducted on printed circuit boards (PCBs) sourced from random access memory (RAM), graphics cards, and motherboards. Each PCB type contained varying amounts of metal, with the ICP-MS analysis revealing significantly higher concentrations of copper (Cu) and tin (Sn) in the graphics card compared to other metals (Table S2). Although tin, a major component in solder used for connecting electronic components to PCBs,^42^ is often overlooked in many studies,^18,44−51^ it has been reported that high concentrations of tin can adversely affect the adsorption efficiency of precious metal recovery polymers.^17,19,20,52^ To mitigate this issue, several studies have proposed two-stage leaching processes^42,53−55^ which selectively remove metals like tin and copper, preventing them from interfering with the adsorption of valuable metals.

In this study, a two-stage leaching method was developed to counteract the negative impact of high tin concentrations on the adsorption efficiency. This method involved using solutions of varying acidity to selectively dissolve less valuable metals, such as tin and copper, while preserving precious metals, such as gold, in the first stage. After the initial leaching process, the remaining PCB fragments were filtered and subjected to a second leaching step using a higher acidity solution to dissolve the gold and other valuable elements into the solution (Figure S4). Prior to leaching, the PCBs were immersed in a 10 M sodium hydroxide (NaOH) solution for 24 h to remove the epoxy coating that protects the metal surfaces. Samples were collected during this process, and ICP-MS analysis was conducted to quantify the amount of metal transferred into the NaOH solution for each PCB type (RAM, graphics card, motherboard), as detailed in Table S3.

Following the epoxy removal, the PCB fragments were washed with tap and deionized water and then subjected to the first-stage leaching process. In this stage, the PCB pieces were immersed in a 1.0 M hydrochloric acid (HCl) solution at a ratio of 100 mL per part, for a total of 400 mL, and agitated in an incubator shaker at 20 °C and 150 rpm for 48 h. After the leaching process, the PCBs were removed and the solution was filtered. The pH of the filtrate was adjusted to pH 2 using a 10 M KOH solution, causing tin to precipitate as a white solid. The types and quantities of metal ions in this leaching solution were analyzed using ICP-MS (Table S4). In the second-stage leaching, the PCB fragments were immersed in a 12 M HCl solution for 24 h under the same agitation conditions. After filtration, the acidic solution was diluted with NaOH and deionized water to a final volume of 1000 mL, maintaining a pH of 2. ICP-MS analysis was conducted to determine the types and concentrations of metal ions present in the second leaching solution (Table S5).

**Figure 7:**
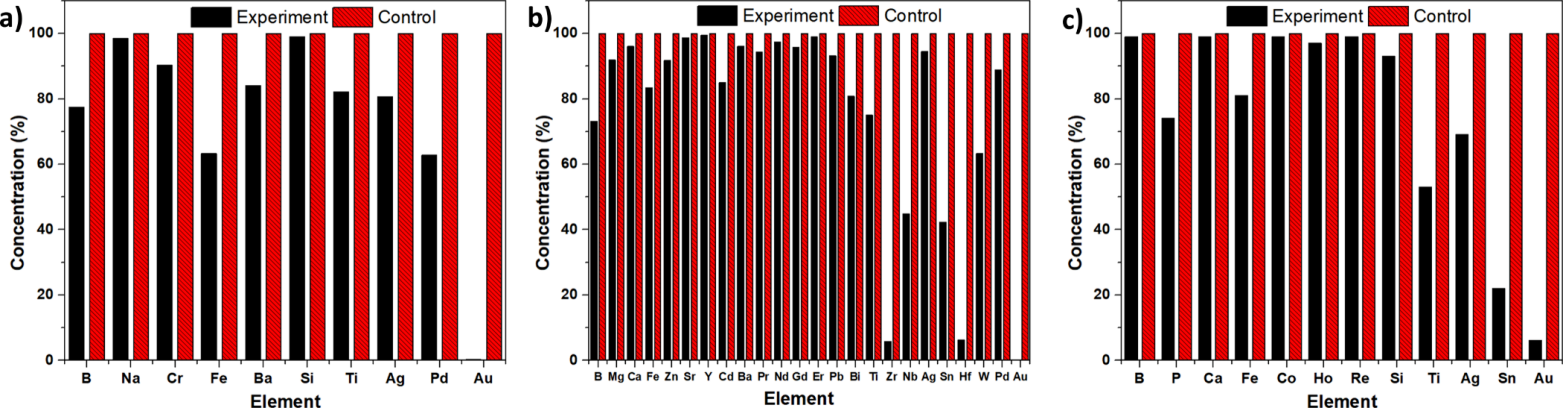
(a) Gold recovery from actual e-waste RAM using EBE-06. (b) Gold recovery from actual e-waste graphic card using EBE-06. (c) Gold recovery from actual e-waste motherboard using EBE-06.

Prior to adsorption experiments, ICP-MS analysis of the leaching solutions revealed that the gold ion concentrations in the PCBs were 389 ppm for RAM, 138 ppm for the graphics card, and 41 ppm for the motherboard. For each adsorption experiment, a 250 mL portion of the solution was combined with 62.5 mg of EBE-06, corresponding to a ratio of 1 mg of polymer per 4 mL of solution. The mixtures were stirred at 20 °C and 120 rpm for 48 h in an incubator shaker. After adsorption, the solutions were filtered to recover the polymer. The results demonstrated that EBE-06 exhibited 99% adsorption efficiency for gold ions from the RAM solution (Figure 7a) and 99% efficiency for gold ions from the graphics card solution (Figure 7b). In the case of the motherboard solution, EBE-06 adsorbed 94% of the available gold ions despite the relatively lower gold content compared to other metals in the solution (Figure 7c). The high selectivity of EBE-06 for gold ions, even in the presence of competing metal ions, confirmed its effectiveness as an adsorbent in the leaching solutions from electronic waste.

Studies have explored various hydrometallurgical methods and solvent extraction techniques for the recovery of precious metals, not only from standard PCBs but also from other forms of e-waste such as mobile phone circuit boards and central processing units (CPUs). These methods, including the use of polymers like EBE-06, have demonstrated the versatility of recovery techniques such as two-stage leaching and selective adsorption, highlighting the potential for broader applications across different PCB waste types.^56−60^

## 4 Conclusions

The carbazole–phosphazene-based polymer EBE-06 was systematically investigated for its gold and precious metal capturing properties. The polymer material (EBE-06) exhibited a high level of selectivity, particularly for gold ions and other valuable metal elements, such as platinum, palladium, and zirconium. This selectivity can be attributed to the high surface area of material, its porosity, and the significant amount of nitrogen heteroatoms. Remarkably, even after three successive uses, the polymer sustained its efficiency in capturing gold at a nearly consistent level. Furthermore, practical applications were performed to assess the selectivity of materials to extract gold and other valuable elements from actual e-waste, using three different PCB materials for analysis.

The gold and precious metal capturing properties of carbazole–phosphazene-based polymer EBE-06 were systematically investigated. The polymer material (EBE-06) demonstrated a high degree of selectivity, particularly for gold ions and other valuable metal elements, such as platinum, palladium, and zirconium. This selectivity is attributable to the material’s high surface area, porosity, and significant nitrogen heteroatom content. Notably, the polymer maintained its efficiency in capturing gold at a nearly consistent level, even after three successive cycles of use. Furthermore, practical applications were conducted to evaluate the material’s selective ability to extract gold and other valuable elements from actual e-waste, utilizing three different types of PCB materials for analysis.

## Data Availability

All underlying data are available in the article itself and its Supporting Information.

## Supporting Information Available

Experimental part (materials and methods, synthesis), ICP study, SEM images, TEM images, EDX images and spectra, adsorption efficiency figures, and metal adsorption and amount tables (PDF)

## Notes

TUBITAK has filed provisional patent applications (1018P101US, TR2023/007409) related to the material and precious metal recovery reported in this article.
