# Supplementary material for: Carbazole–Phosphazene Based Polymer for Efficient Extraction of Gold and Precious Elements from Electronic Waste
Source: ACS Omega. 2024 Nov 18;9(48):47884–92. doi: 10.1021/acsomega.4c09068 (PMC11618425; doi:10.1021/acsomega.4c09068)
Supplement: Supplementary file 1 — ao4c09068_si_001.pdf [file ao4c09068_si_001.pdf]

Supporting Information

Carbazole−Phosphazene Based Polymer for Efficient Extraction of Gold and Precious Elements from Electronic Waste

Evren CUCU[1,2], Betul ARI ENGIN[1], Murat TUNC[1], Ramazan ALTUNDAŞ[2]

and Ali Enis SADAK[1]*

[1]TUBITAK UME, Chemistry Group Laboratories, 41470, Gebze, Kocaeli, Türkiye

[2]Gebze Technical University, Department of Chemistry, 41400, Gebze, Kocaeli, Türkiye

E-mail: alienis.sadak@tubitak.gov.tr (A.E.S

Contents **Page**

1. General methods, Characterization 3
   1.1 General Methods and Characterization 3
   1.2 Synthesis of EBE-06 3
2. General Spectra 4
3. ICP Study 6
4. Demonstration of precious metal capture from actual PCB e-waste 12
5. SEM, TEM EDX images of EBE-06 13
6. References 24

Figure List **Page**

Figure S1. Time-dependent platinum adsorption efficiencies at varying pH values. 4
Figure S2. Time-dependent silver adsorption efficiencies at varying pH values. 4
Figure S3. Time-dependent palladium adsorption efficiencies at varying pH values. 5
Figure S4. Total process of precious metal capture from actual PCB e-waste 12
Figure S5. Scanning Electron Microscopy (SEM) Images of EBE-06. 15
Figure S6. Energy Dispersive X-ray (EDX) Images of EBE-06. 16
Figure S7. Transmission Electron Microscopy (TEM) Images of EBE-06 Au Adsorption. 18
Figure S8. Energy Dispersive X-ray (EDX) Images of EBE-06 Au Adsorption. 21
Figure S9. Energy Dispersive X-ray (EDX) Images of EBE-06 Au Desorption. 24

Table List **Page**

Table S1. Metal adsorption selectivity of EBE-06. 6
Table S2. Metal amounts of in the graphic cards (ppb). 7
Table S3. Metal amounts of PCBs from NaOH solutions (ppb). 8
Table S4. Metal amounts of PCBs from first leaching experiment (ppb). 9
Table S5. Metal amounts of PCBs from second leaching experiment (ppb). 10

1. General methods, Characterization

1.1 General Methods and Characterization

Gold and other metal concentrations measurements in solution were performed using HR-Thermo Element-2 instrument inductively coupled plasma mass spectrometry (ICP-MS). Surface area analyzes of EBE-06 were performed with Brunauer-Emmett-Teller (BET) at P/P_o = 0.01-0.10 and Langmuir methods at P = 30-220 mbar. X-ray photoelectron spectroscopy (XPS) analysis was performed by Thermo Scientific Model K-Alpha XPS instrument using monochromatic Al Kα radiation (1486.7 eV). Survey spectra and high resolution spectra were acquiredusing analyzer pass energies of 50 eV. Powder X-ray diffraction (XRD) results were obtained by Shimadzu XRD 6000 operated at 40 kV and 40 mA with Cu Kα radiation (step size: 0.020, step time: 0.60 s). Using Cu Kα radiation (k = 1.5406 Å) with scattering angles (2h) of 30–80°. Scanning & Transmission Electron Microscopy (SEM & STEM) and Energy dispersive X-ray (EDX) images were obtained Hitachi SU-5000 microscope worked at changing voltage of 5.0 – 30.0 kV.

1.2 Synthesis of EBE-06

Anhydrous $AlCl_3$ (2.79 g, 20.92 mmol, 12 equiv.) was added in 40 mL of 1,2-Dichlorobenzene at room temperature and this mixture stirred for 15 min. Then hexachlorocyclotriphosphazene (606 mg, 1.74 mmol, 1 equiv.) in 15mL of 1,2-Dichlorobenzene and 1,3,5-tri(9*H*-carbazol-9-yl) benzene (1.00 g, 1.74 mmol, 1 equiv.) in 20 mL of 1,2-Dichlorobenzene were added to this mixture and stirred for 6 h at room temperature. After 6 h, it was heated to 190 ^oC and stirred for 18 h. The reaction mixture was cooled to room temperature. The black solids were filtered through No.3 glass filter and washed subsequently with 200 mL of 2 N HCl, 200 mL of distilled water, 100mL of methanol. The crude material in 100 mL of methanol was sonicated for 30 minutes and it was filtered and solid was sequentially extracted by Soxhlet with methanol, tetrahydrofuran, and acetone (100 mL for each cycle) for 24 hours. After the washing processes were completed, the material was dried in a vacuum oven at 50 ^oC for 6 h, at 70 ^oC for 6 h and at 120 ^oC for 24 h. The BE-04 was obtained as a brown solid in 1.198 g, 98% yield.
IR (ATR, powder): 1590, 1454, 1311, 1214, 1056, 867, 803, 589, 541 cm^{-1} [1].

2. General Spectra

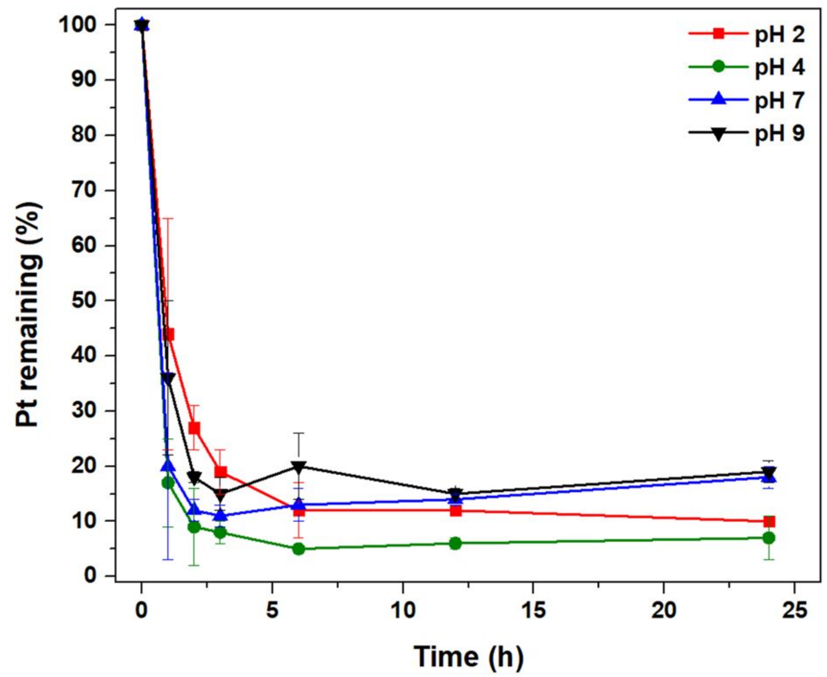

Figure S1. Time-dependent platinum adsorption efficiencies at varying pH values.

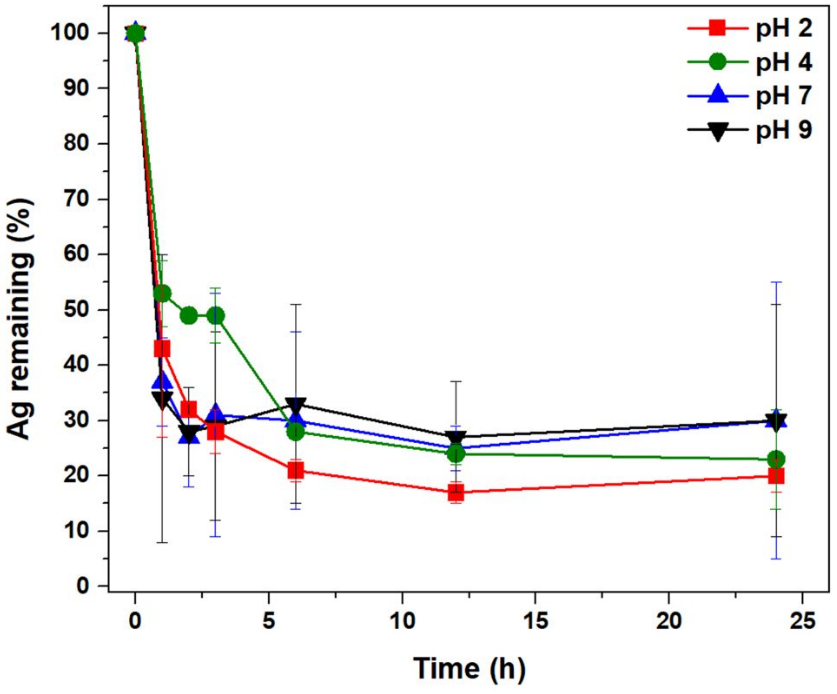

Figure S2. Time-dependent silver adsorption efficiencies at varying pH values.

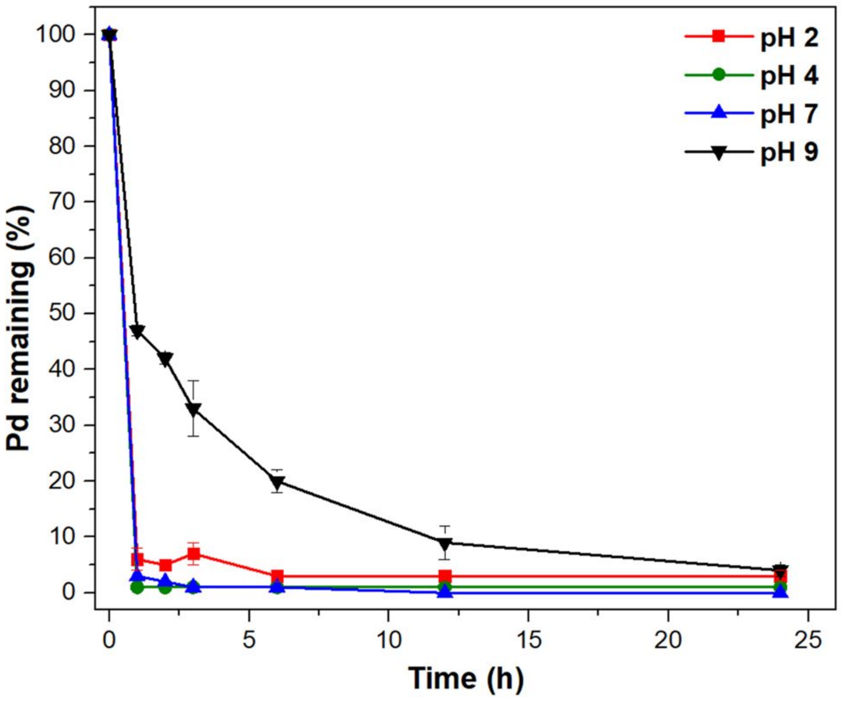

Figure S3. Time-dependent palladium adsorption efficiencies at varying pH values.

3. ICP Study

Table S1. Metal adsorption selectivity of EBE-06.

Element	% Adsorption	Element	% Adsorption
Li	0	Gd	0,49
Be	0	Tb	1,41
B	0	Dy	1,07
Na	0	Ho	0,35
Mg	0	Er	0,94
Al	0	Tm	0,67
P	0	Yb	0,77
K	0	Lu	0,95
Ca	0	Re	34,99
Sc	98,63	Tl	60,48
V	1,32	Pb	0,75
Cr	0	Bi	34,46
Mn	0	Th	81,79
Fe	40,74	U	81,8
Co	0	Si	0
Ni	0	Ti	59,84
Cu	0	Ge	5,23
Zn	0	Zr	96,77
Ga	7,62	Nb	1,94
As	95,88	Mo	76,09
Se	88,43	Ag	44,95
Rb	0,03	Sn	83,42
Sr	0	Sb	0
Y	0,17	Te	44,89
Cd	0,61	Hf	96,44
In	4,89	Ta	60,85
Cs	0	W	53,93
Ba	0	Ru	0,99
La	0	Rh	0
Ce	0,67	Pd	98,18
Pr	0	Os	23,18
Nd	0	Ir	3,71
Sm	1,43	Pt	44,99
Eu	1,45	Au	98,47

Table S2. Metal amounts of in the graphic cards **(**ppb**).**

No	Metal	Metal amount (ppb)
1	Na	22744
2	Mg	21406
3	Al	96583
4	Cr	3306
5	Mn	11813
6	Fe	1610278
7	Co	20758
8	Ni	1584211
9	Cu	38815120
10	Zn	1231819
11	Ga	34
12	As	1317
13	Rb	6
14	Sr	3037
15	Y	7167
16	Cd	11
17	Ba	198495
18	Pb	748737
19	Ti	67340
20	Zr	3401
21	Mo	568
22	Ag	15471
23	Sn	15634751
24	Sb	5935
25	Te	8302
26	W	2561
27	Pd	4102
28	Pt	6
29	Au	1223

Table S3. Metal amounts of PCBs from NaOH solutions (ppb).

Element	RAM	Graphic card	Motherboard
Li	0	128	23
B	54431	61215	21241
Na	0	1601996	0
Mg	3470	4066	1907
Al	200137	59370	2447918
P	145	1829	4620
Ca	21167	43028	21034
Cr	223	0	0
Mn	166	248	210
Fe	5530	20805	4219
Ni	275	148	0
Cu	1315439	2917	257
Zn	43632	20019	16792
Se	339	238	316
Sr	6296	3929	7069
Ba	283353	151574	408345
Eu	148	85	149
Pb	1911	815	0
Si	403021	555552	138642
Mo	0	288	0
Ag	196828	0	0
Sn	3646	25927	64644
Sb	8656	2427	0

Table S4. Metal amounts of PCBs from first leaching experiment (ppb).

Element	RAM	Graphic card	Motherboard
Li	0	128	548
B	2821	26042	88525
Na	0	311291875	34912
Mg	2056	15991	33117
Al	8558	73479	372272
P	311	0	3448
Ca	22182	113280	608253
Cr	898	1758	851
Mn	1770	20844	17752
Fe	38200	5867457	155401
Co	1037	37029	17673
Ni	321400	8282432	4269739
Cu	1315439	0	116629880
Zn	4877	573535	18330901
As	0	4522	0
Se	0	913646	0
Rb	0	205	517
Sr	384	2791	9793
Y	895	7157	13461
In	120	104	126
Ba	64824	248360	593031
La	0	0	116
Ce	0	0	268
Nd	0	493	0
Sm	0	0	118
Eu	0	0	264
Gd	0	154	676
Dy	953	0	1250
Ho	0	0	1048
Pb	60287	4453011	17449
Bi	178	0	196
Si	985	5651	3926
Ti	0	328	0
Ge	0	1326	0
Mo	0	320	157
Ag	196828,1	132663	1594187

Sn	0	82686	958
Pd	2507	1467	2530

Table S5. Metal amounts of PCBs from second leaching experiment **(**ppb**).**

Element	RAM	Graphic card	Motherboard
B	167	1880	16031
Na	0	12435077	0
Mg	0	1150	9615
Al	0	2371	108238
P	0	368	70065
Ca	0	40588	132452
Cr	131	614	2833
Mn	0	3753	20623
Fe	490	105913	69806
Co	0	1014	22442
Ni	120	47638	1165190
Cu	196	138732	55162588
Zn	0	36717	1062192
As	0	443	0
Rb	0	0	1008
Sr	0	31073	7605
Y	0	3521	4834
Cd	0	475	0
Ba	1527	1738723	368749
Pr	0	290	0
Nd	0	13870	0
Sm	0	375	0
Eu	0	628	207
Gd	0	1609	267
Dy	0	0	7904
Ho	0	0	5052
Er	0	121	0
Pb	0	0	56373
Bi	0	4879	795
Si	1361	6871	3285
Ti	302	407474	17150

Zr	0	150156	0
Nb	0	464	0
Mo	0	287	0
Ag	25843	33697	945665
Sn	0	171437	5532
Sb	0	939	0
Hf	0	3417	0
W	0	331	0
Ru	0	0	115
Pd	722	6159	1345
Pt	0	141	0
Au	389734	138638	41573

4. Demonstration of precious metal capture from actual PCB e-waste

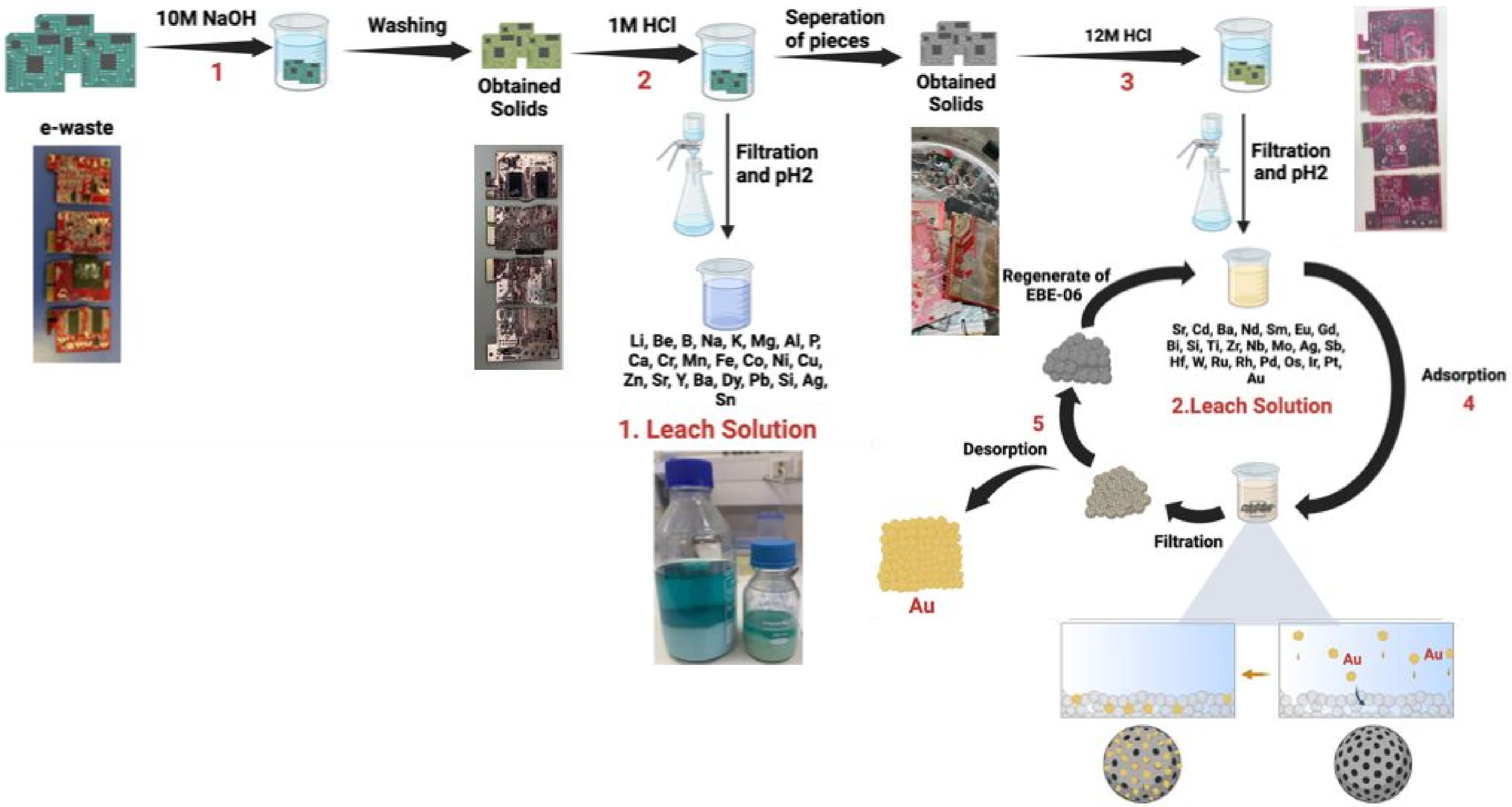

Figure S4. Total process of precious metal capture from actual PCB e-waste

5. SEM, TEM EDX images of EBE-06

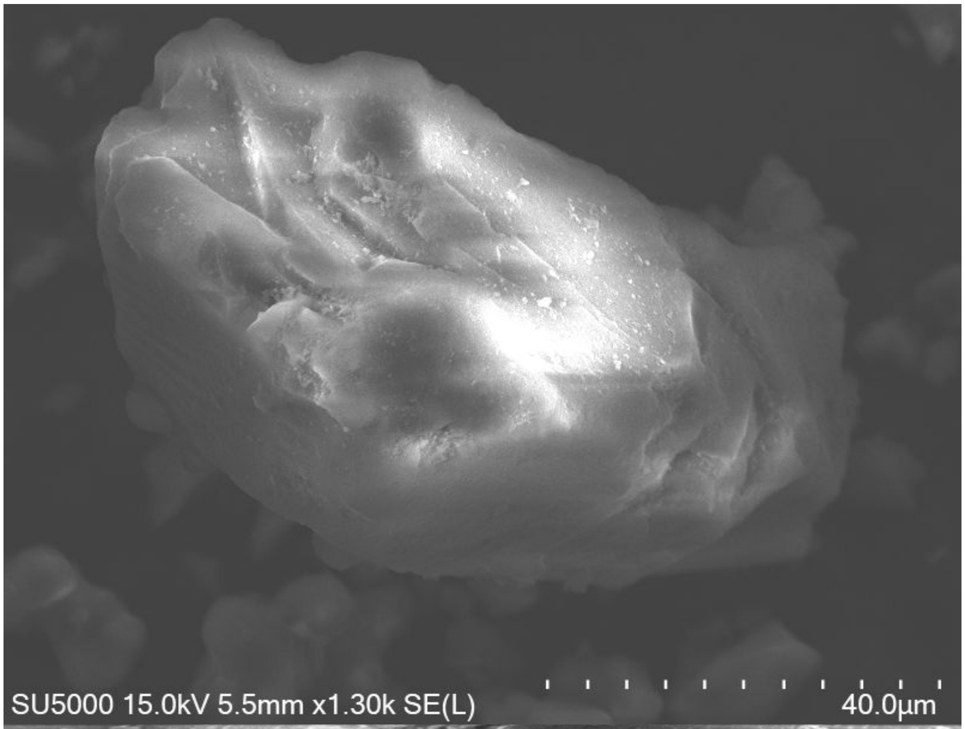

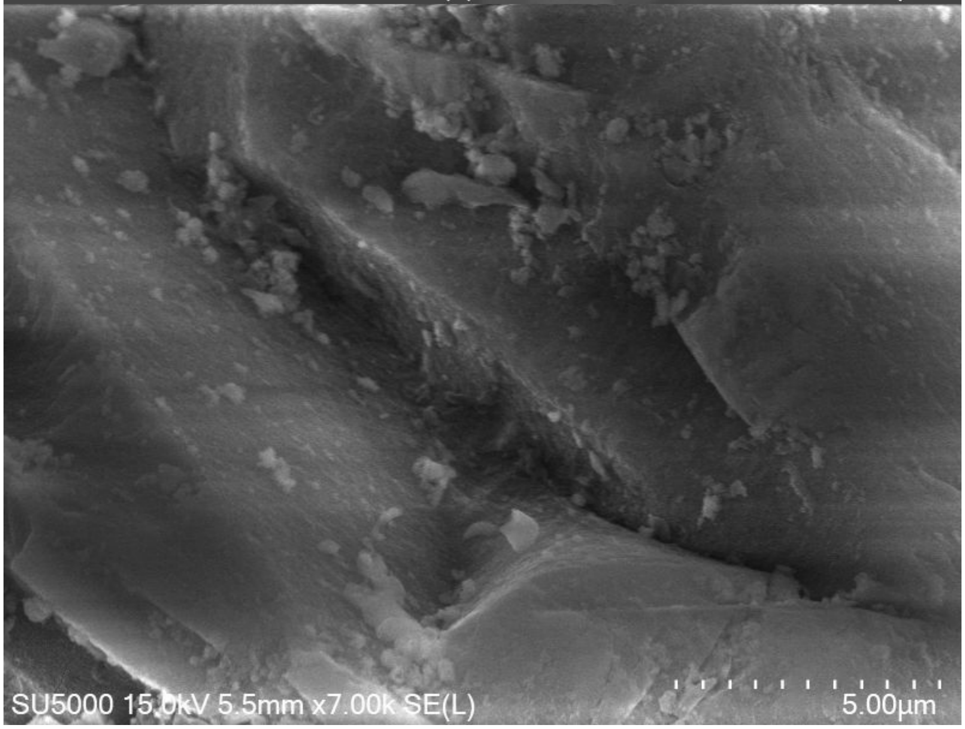

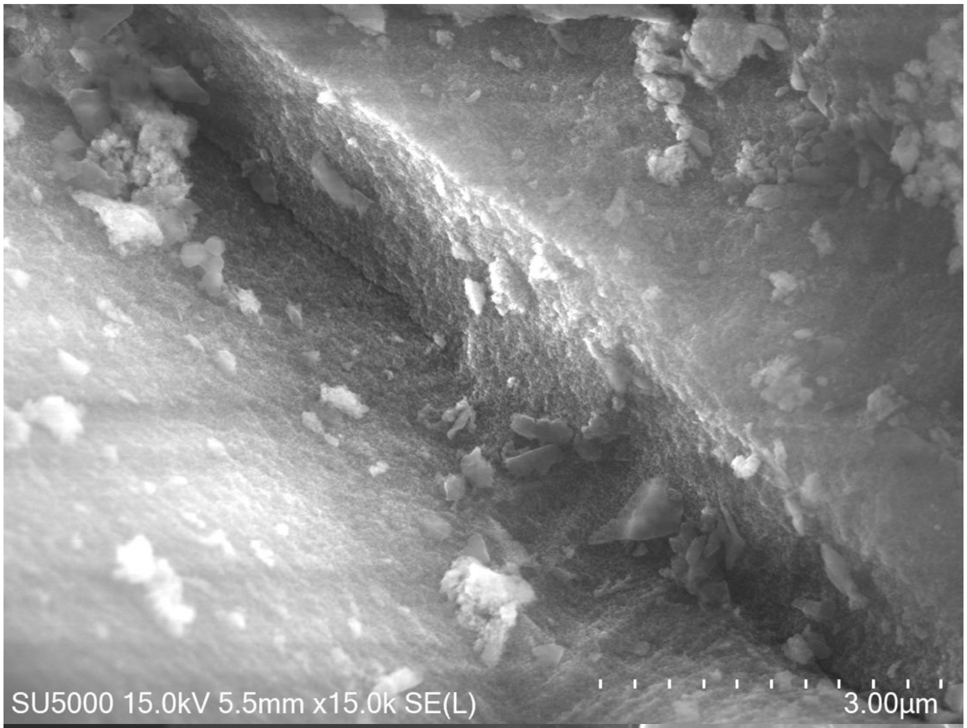
SU5000 15.0kV 5.5mm x15.0k SE(L)
3.00µm

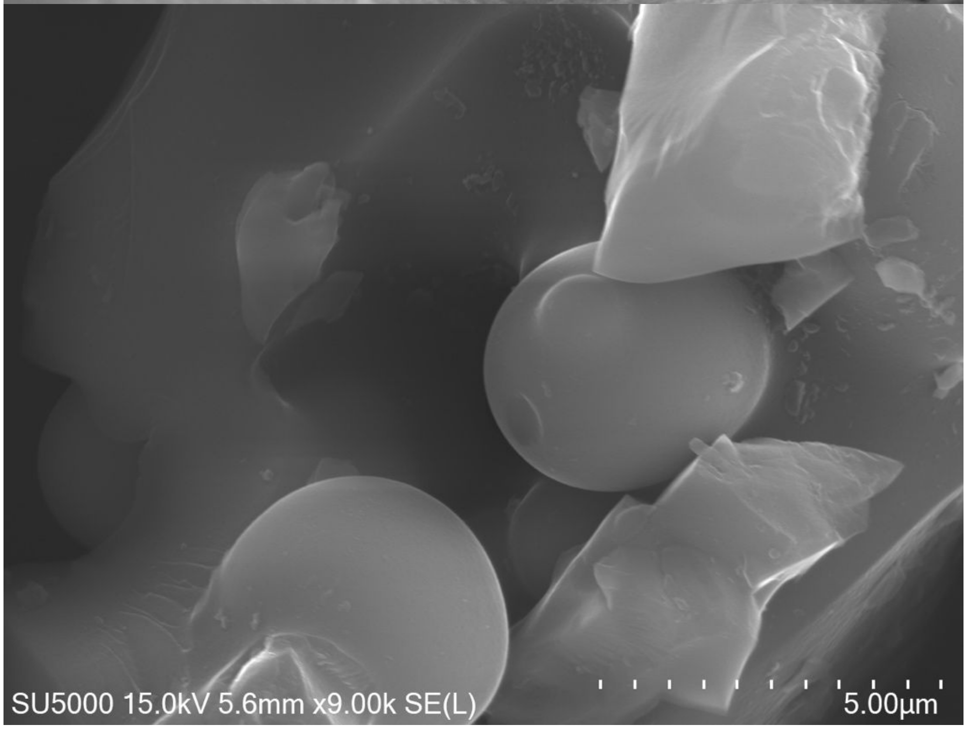
SU5000 15.0kV 5.6mm x9.00k SE(L)
5.00µm

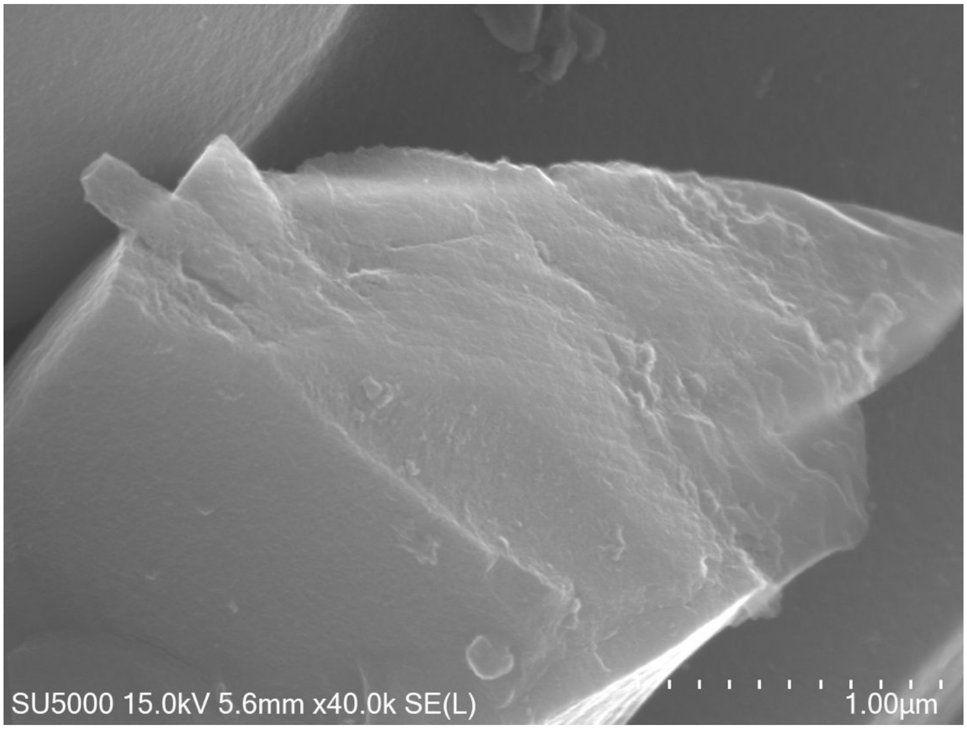

Figure S5. Scanning Electron Microscopy (SEM) Images of EBE-06.

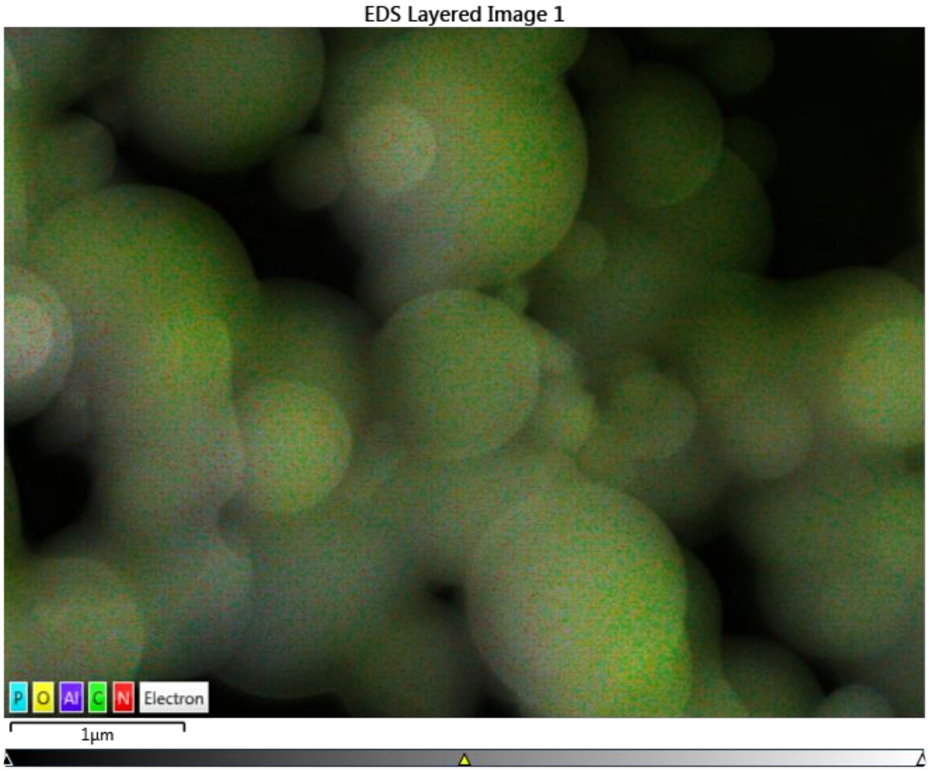

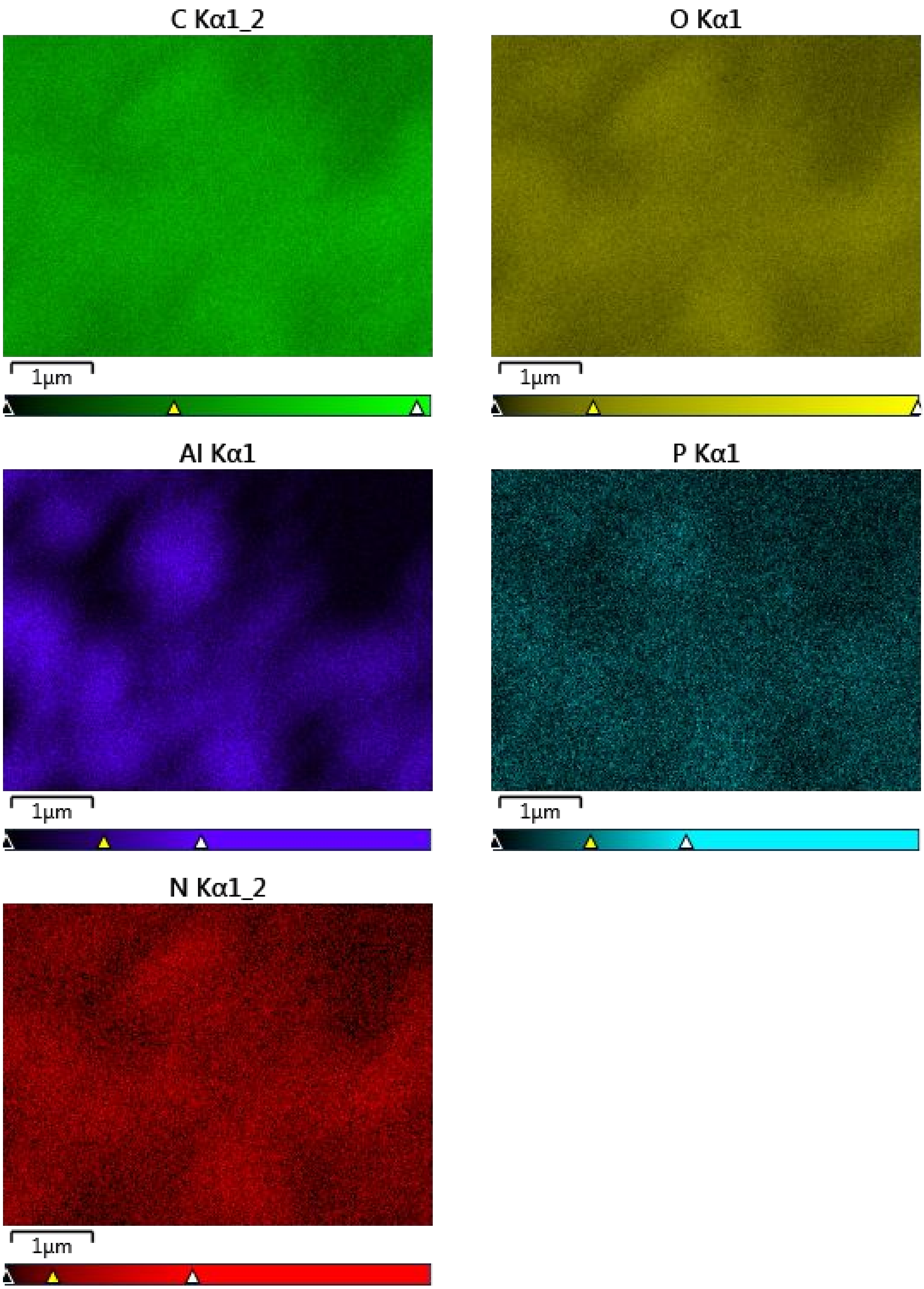

Figure S6. Energy Dispersive X-ray (EDX) Images of EBE-06.

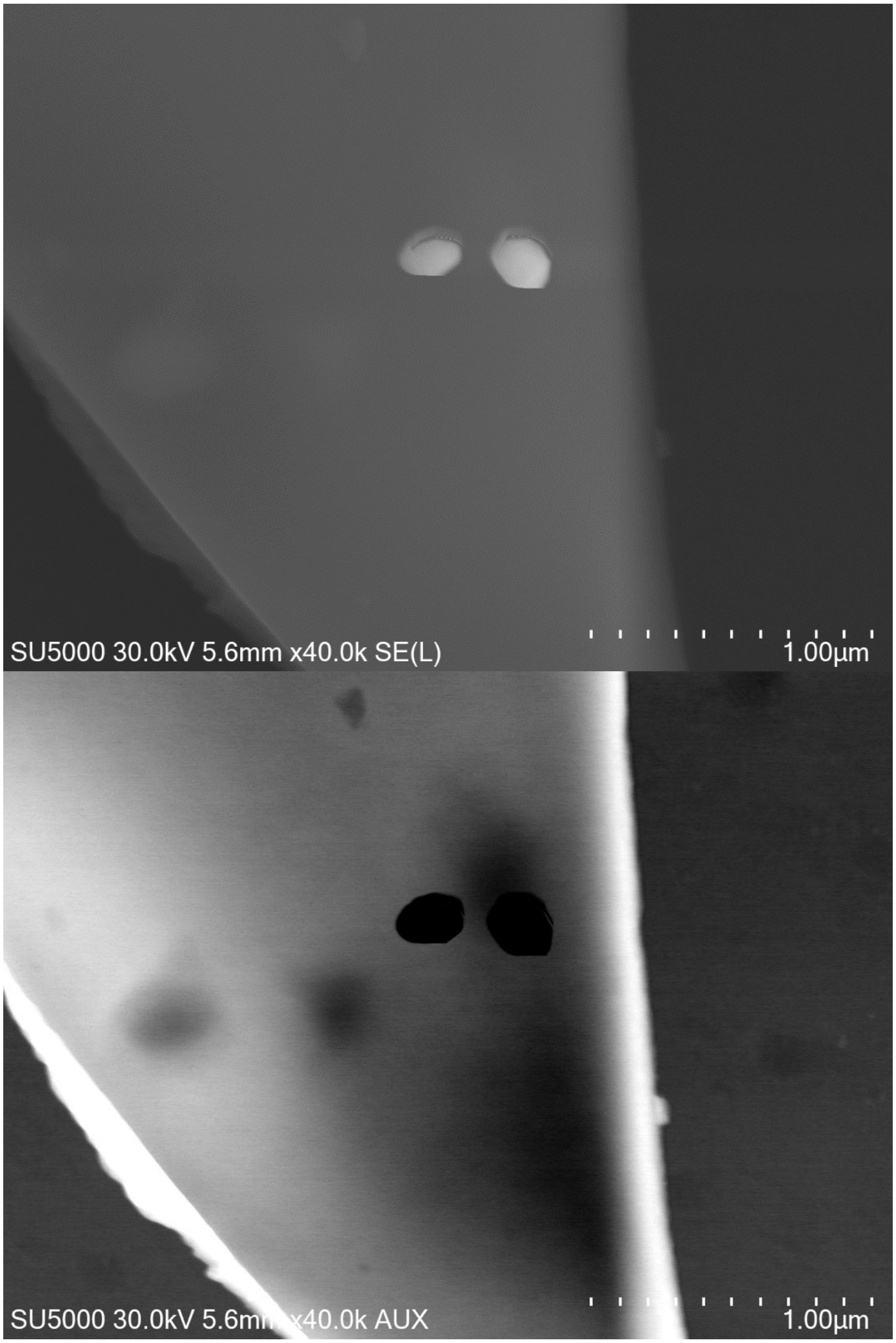
SU5000 30.0kV 5.6mm x40.0k SE(L)
1.00µm
SU5000 30.0kV 5.6mm x40.0k AUX
1.00µm

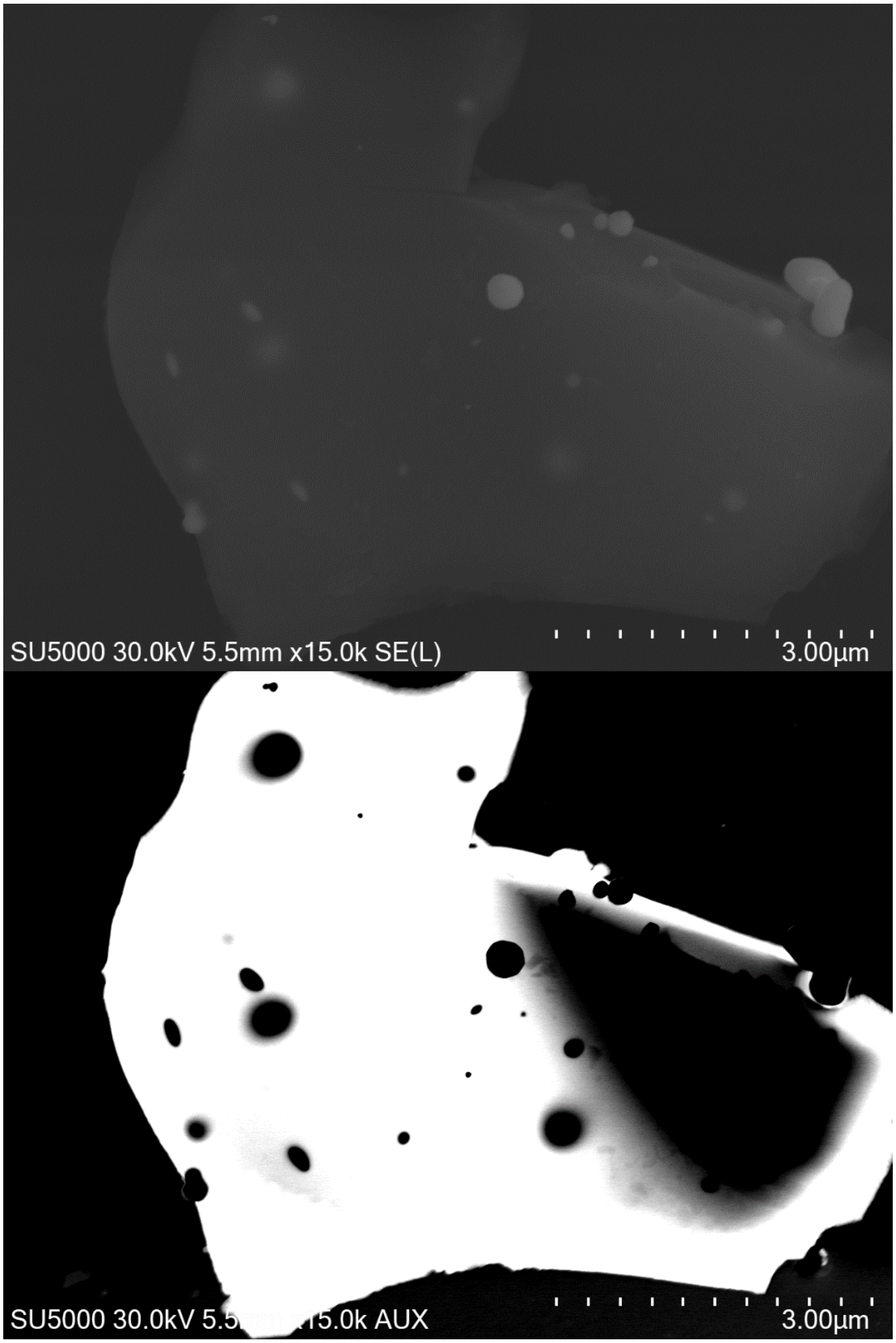

Figure S7. Transmission Electron Microscopy (TEM) Images of EBE-06 Au Adsorption.

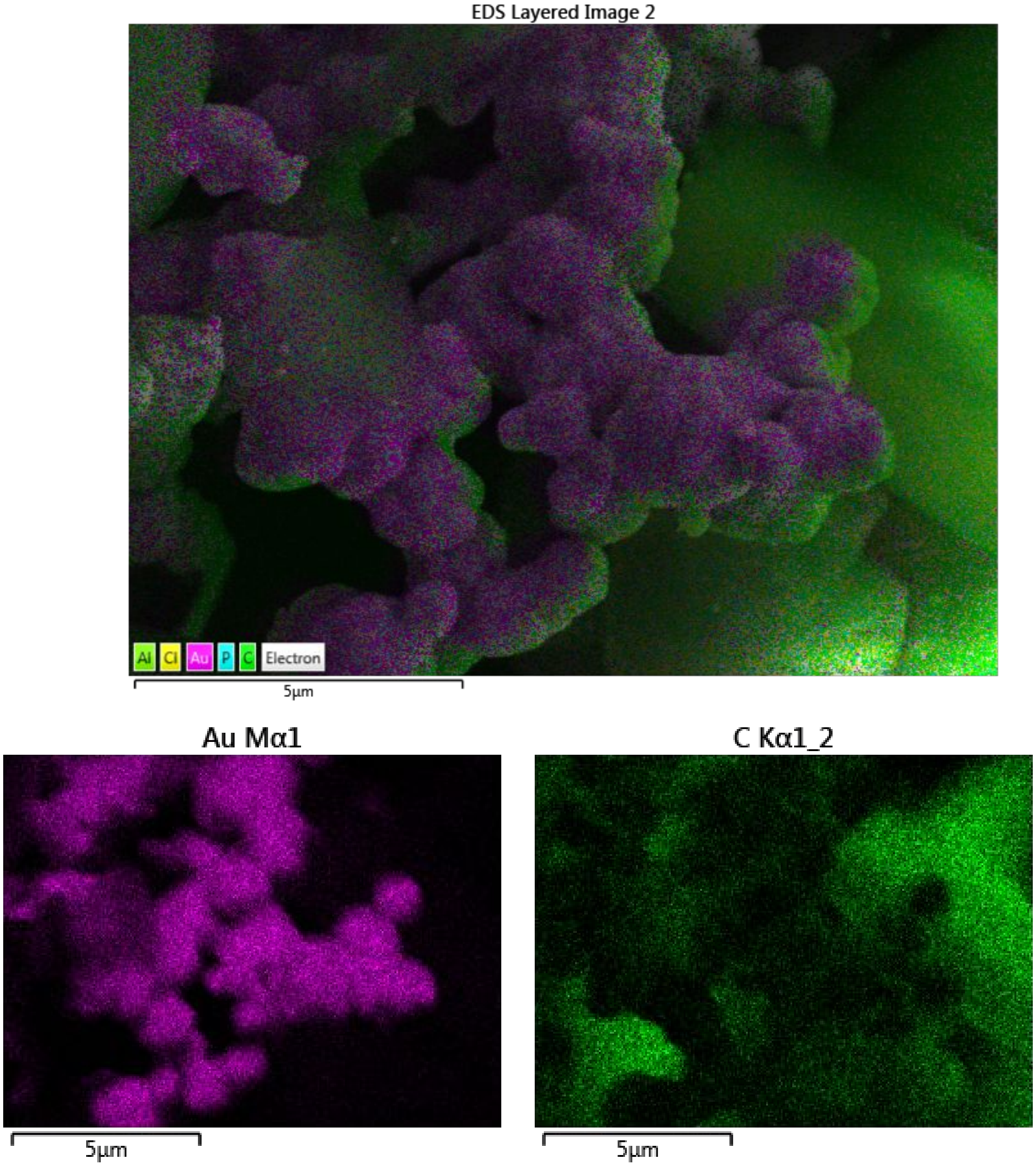
EDS Layered Image 2
Al
Cl
Au
P
C
Electron
5µm
Au Mα1
5µm
C Kα1_2
5µm

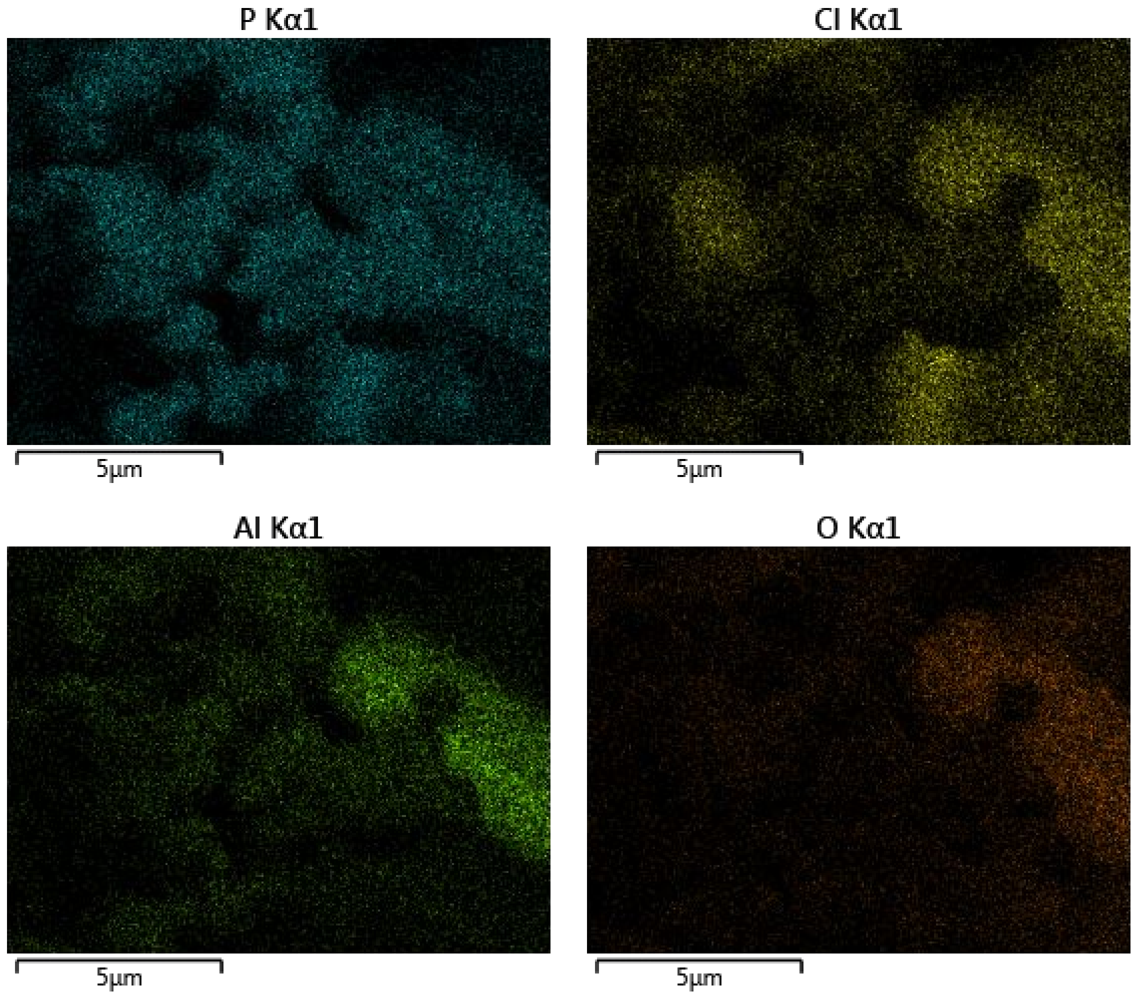
P Kα1
5µm
Cl Kα1
5µm
Al Kα1
5µm
O Kα1
5µm

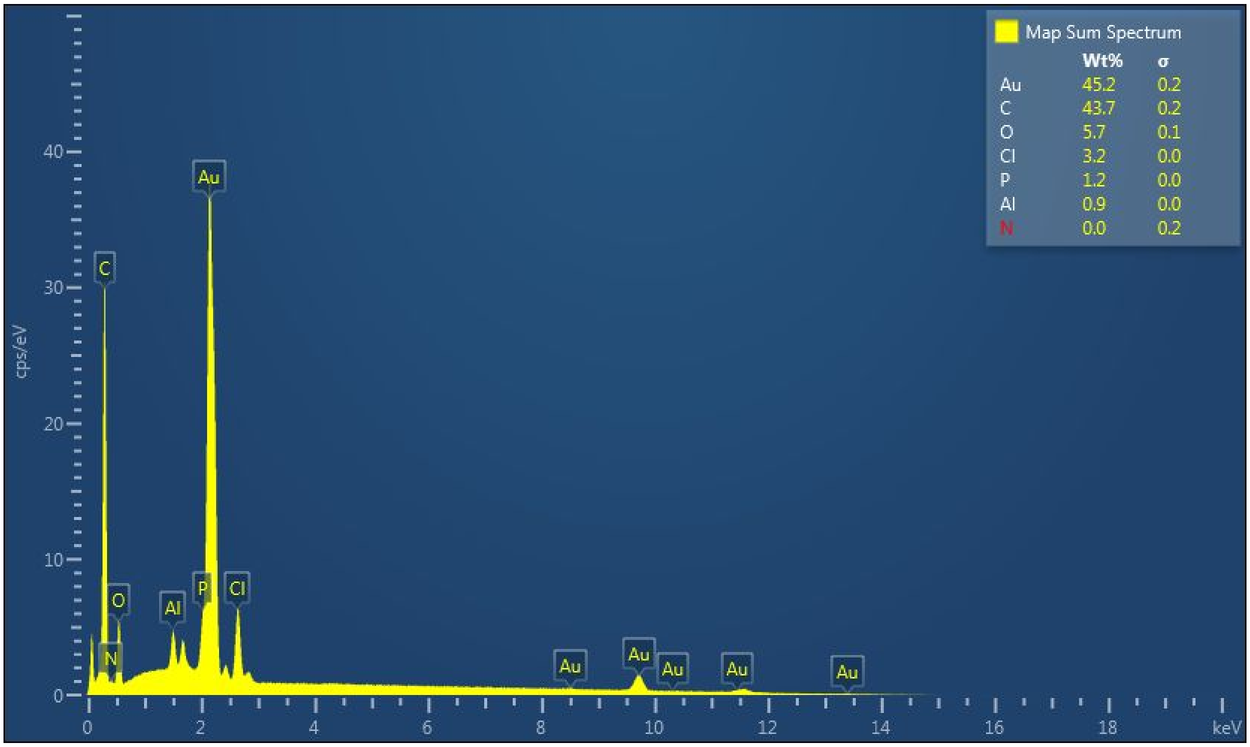

Figure S8. Energy Dispersive X-ray (EDX) Images of EBE-06 Au Adsorption.

EDS Layered Image 3

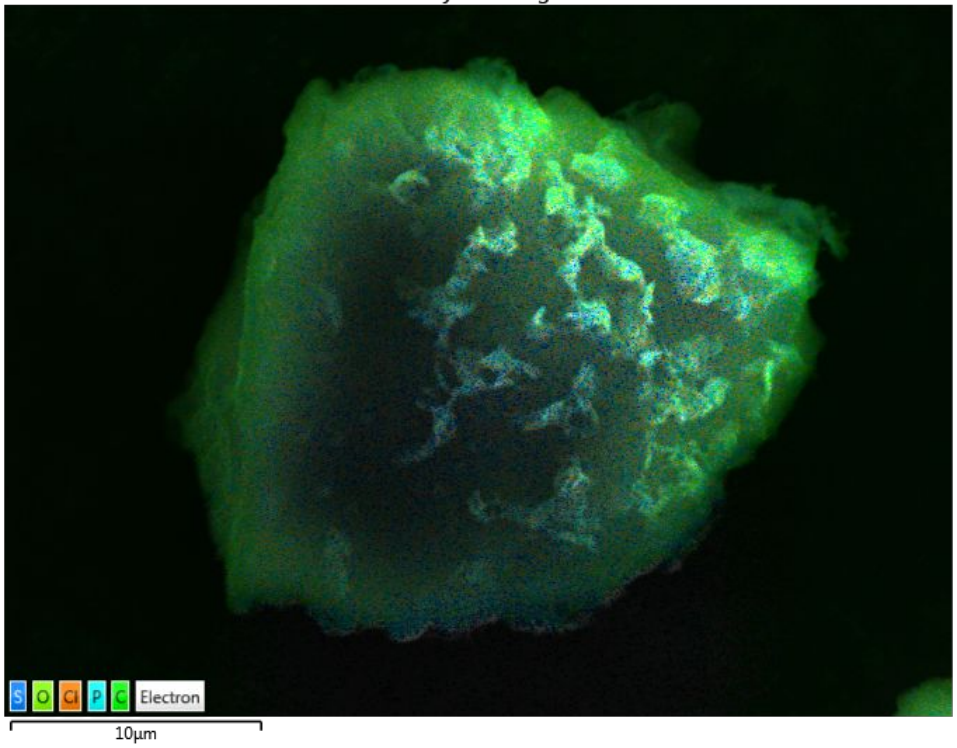

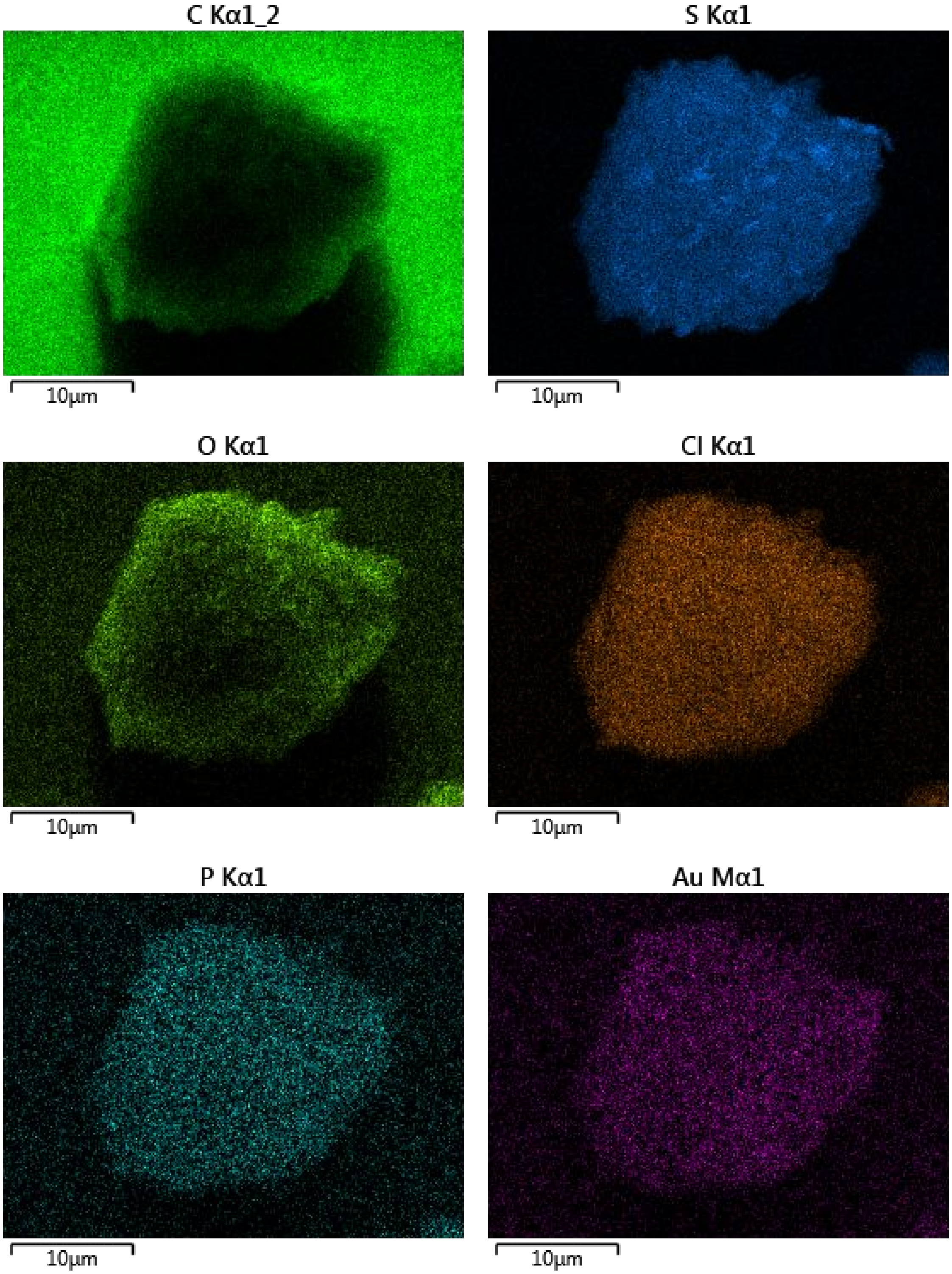
C Kα1_2
10μm
S Kα1
10μm
O Kα1
10μm
Cl Kα1
10μm
P Kα1
10μm
Au Mα1
10μm

Al Kα1

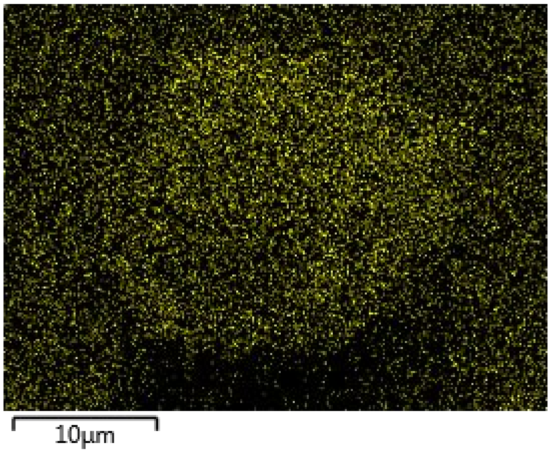

Si Kα1

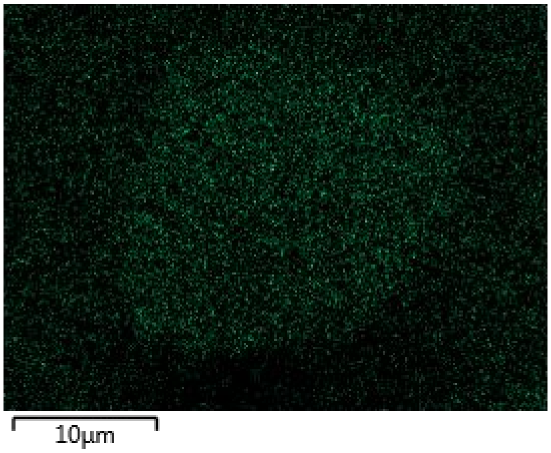

N Kα1_2

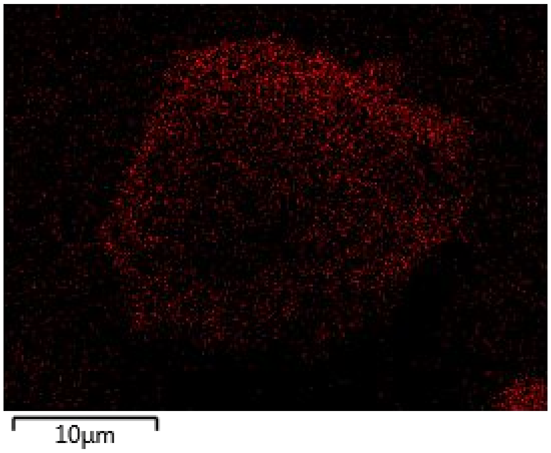

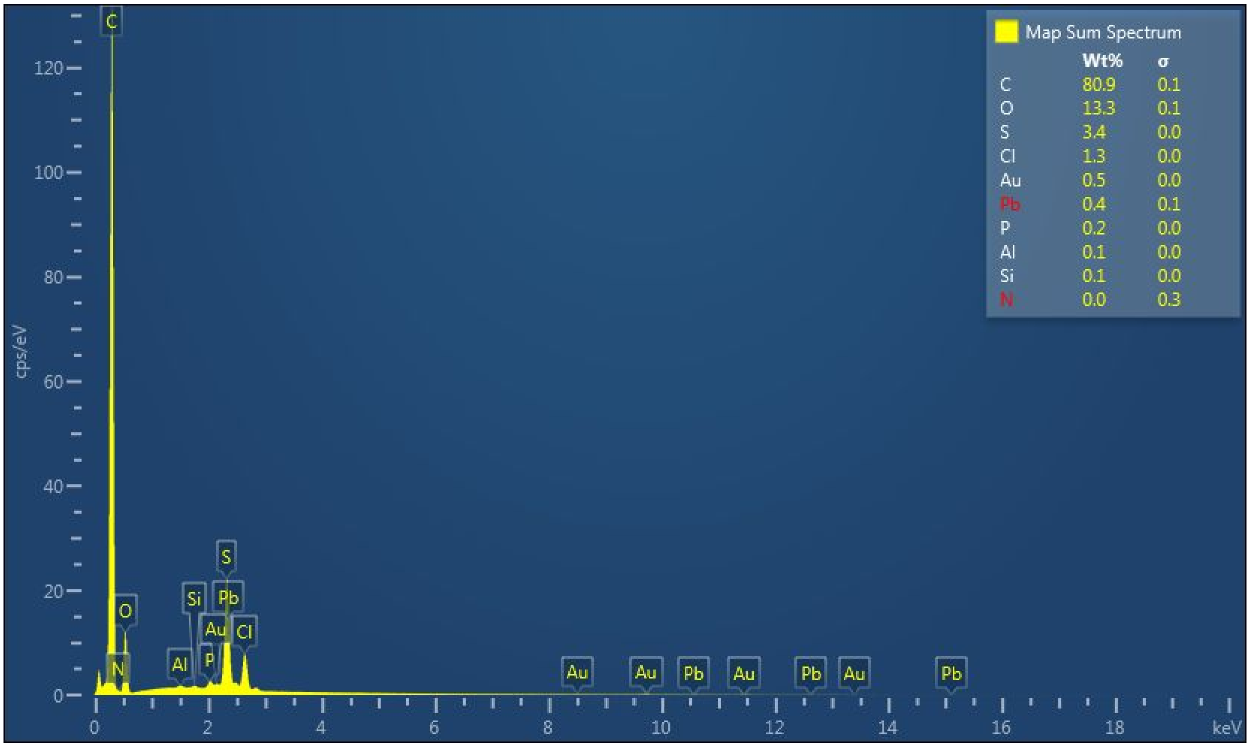

Figure S9. Energy Dispersive X-ray (EDX) Images of EBE-06 Au Desorption.

6. References

1. Sadak, A. E., Cucu, E., Hamur, B., Ün, İ., & Altundas, R. (2023). Cyclotriphosphazene and tricarbazole based microporous hyper-crosslinked conjugated polymer for CCUS: Exceptional CO2 selectivity and high capacity CO2, CH4, and H2 capture. *Journal of CO2 Utilization*, *67*, 102304.
